# Autophagy is deregulated in cancer-associated fibroblasts from oral cancer and is stimulated during the induction of fibroblast senescence by TGF-β1

**DOI:** 10.1038/s41598-020-79789-8

**Published:** 2021-01-12

**Authors:** May Leng Tan, E. Kenneth Parkinson, Lee Fah Yap, Ian C. Paterson

**Affiliations:** 1 Department of Oral and Craniofacial Sciences, Level 9, Postgraduate and Research Tower, Faculty of Dentistry, University of Malaya, 50603 Kuala Lumpur, Malaysia; 2 Centre for Immunobiology and Regenerative Medicine, Institute of Dentistry, Barts and the London School of Medicine and Dentistry, Queen Mary University of London, London, UK; 3 Oral Cancer Research & Coordinating Centre, Faculty of Dentistry, University of Malaya, Kuala Lumpur, Malaysia

**Keywords:** Cancer, Cell biology

## Abstract

Many of the characteristics ascribed to cancer-associated fibroblasts (CAFs) are shared by activated, autophagic and senescent fibroblasts. Whilst most oral squamous cell carcinomas (OSCCs) are genetically unstable (GU-OSCC), genetically stable variants (GS-OSCC) have been described and, notably, CAF activation (myofibroblast differentiation) and senescence are characteristics particularly associated with GU-OSCCs. However, it is not known whether autophagy is disrupted in these cells or whether autophagy regulates the development of the myofibroblast and senescent phenotypes. In this study, we show that senescent CAFs from GU-OSCCs contained more autophagosomes than normal human oral fibroblasts (NHOFs) and CAFs from GS-OSCCs possibly due to autophagic impairment. Further, we show that deregulation of autophagy in normal fibroblasts, either by inhibition with autophagy inhibitor, SAR405, or activation with TGF-β1, induced fibroblast activation and senescence: In response to TGF-β1, autophagy was induced prior to the development of the activated and senescent phenotypes. Lastly, we show that both SAR405- and TGF-β1-treated NHOFs enhance OSCC cell migration but only TGF-β1-treated cells increase OSCC invasion through Matrigel, indicating that TGF-β1 has additional effects that are independent of fibroblast activation/senescence. These results suggest a functional role for autophagy in the development of myofibroblast and CAF phenotypes.

## Introduction

Oral squamous cell carcinoma (OSCC) accounts for almost 355,000 new cases annually worldwide and there is an increasing incidence in many countries, particularly Papua New Guinea, the Indian subcontinent and South-East Asian countries^1^. Despite advances in cancer therapy, only approximately 50% of patients survive for 5 years; patient prognosis is poor because of late presentation, locoregional invasion and recurrence, the development of second primary tumours and metastatic disease^2,3^. The molecular pathogenesis of OSCC has been extensively studied and our current understanding is that most tumours are human papillomavirus (HPV) negative genetically unstable OSCCs (GU-OSCC) that harbour a multitude of copy number alterations (CNA) with functional loss of p53 and p16^Ink4a^. A smaller subset of genetically stable OSCCs (GS-OSCC) with a better prognosis has been identified; these tumours contain wild-type p53, are CNA-silent and often contain oncogenic *HRAS* mutations and inactivation of *CASP8*^4–6^. However, it is well recognised that non-malignant components within the tumour microenvironment (TME) also influence tumour development and progression^7,8^. Cancer-associated fibroblasts (CAFs) are often the most abundant stromal cell type and they actively participate in the reciprocal communication between tumour cells and other host cells in the TME to create a tumour-permissive microenvironment in a number of solid tumours, including OSCC^9^.

CAFs react to stress within the tumour environment in a variety of ways that not only allow them to adapt to severely stressful conditions but also enhances pro-tumourigenic behaviour in tumour cells^9^. Such stress responses in CAFs from OSCCs include myofibroblast differentiation (fibroblast activation) and senescence^10^. CAF senescence (permanent proliferative arrest) and activation are characteristics particularly associated with GU-OSCCs compared to GS-OSCCs^11^ and are thought to be tumour-promoting, essentially due to a unique secretome that is collectively known as the senescence-associated secretory phenotype (SASP)^12,13^. However, it is possible that GU- and GS-OSCCs are not entirely distinct entities and tumours may contain mixtures of genetically stable and unstable keratinocytes^14^ (E.K. Parkinson, unpublished data). This concept is supported indirectly by the observation that α-smooth muscle actin expression (α-SMA, a marker of fibroblast activation) can be homogeneous or focal within OSCC tissues^15^. It has been shown previously that keratinocytes from GU-OSCCs induced senescence in fibroblasts via the production of ROS and this mechanism is dependent upon CAF-derived TGF-β1^16^. TGF-β1-induced fibroblast activation has been shown to precede senescence^16^ and activated myofibroblasts share overlapping gene expression profiles with senescent fibroblasts^17^, indicating that these phenotypes are closely related.

Autophagy is a self-catabolic digestion system whereby cell self-digests its own long-lived cytosolic proteins and organelles and is important for homeostasis and as an adaptive response to cellular stress^18,19^. Autophagy is a multi-step, highly dynamic process that is regulated by a unified set of highly conserved autophagy-related genes (*ATG*)^20^. Interestingly, many of the characteristics ascribed to senescent CAFs are also shared by fibroblasts that have been induced to undergo autophagy^21^. Accumulating evidence indicates that aberrant autophagy in stromal fibroblasts might contribute to the acquisition of the senescent phenotype^21–24^. However, this has not been examined in CAFs from OSCCs or in the context of TGF-β1-induced fibroblast activation and senescence.

In this study, we investigated the autophagic and senescent phenotypes in normal fibroblasts and CAFs from GU- and GS-OSCCs. We show that CAFs from GU-OSCCs concomitantly express markers of senescence and contain increased numbers of autophagosomes, indicating that the processes of senescence and autophagy are closely related. Further, we demonstrate that deregulation of autophagy in normal fibroblasts induces both fibroblast activation and senescence. Lastly, we provide evidence that altered autophagy in normal fibroblasts enhanced OSCC cell migration. The results highlight a key role for autophagy in the development of myofibroblast and CAF phenotypes.

## Results

### CAFs from GU-OSCCs demonstrate high levels of senescence and accumulation of autophagosomes

**Figure 1:**
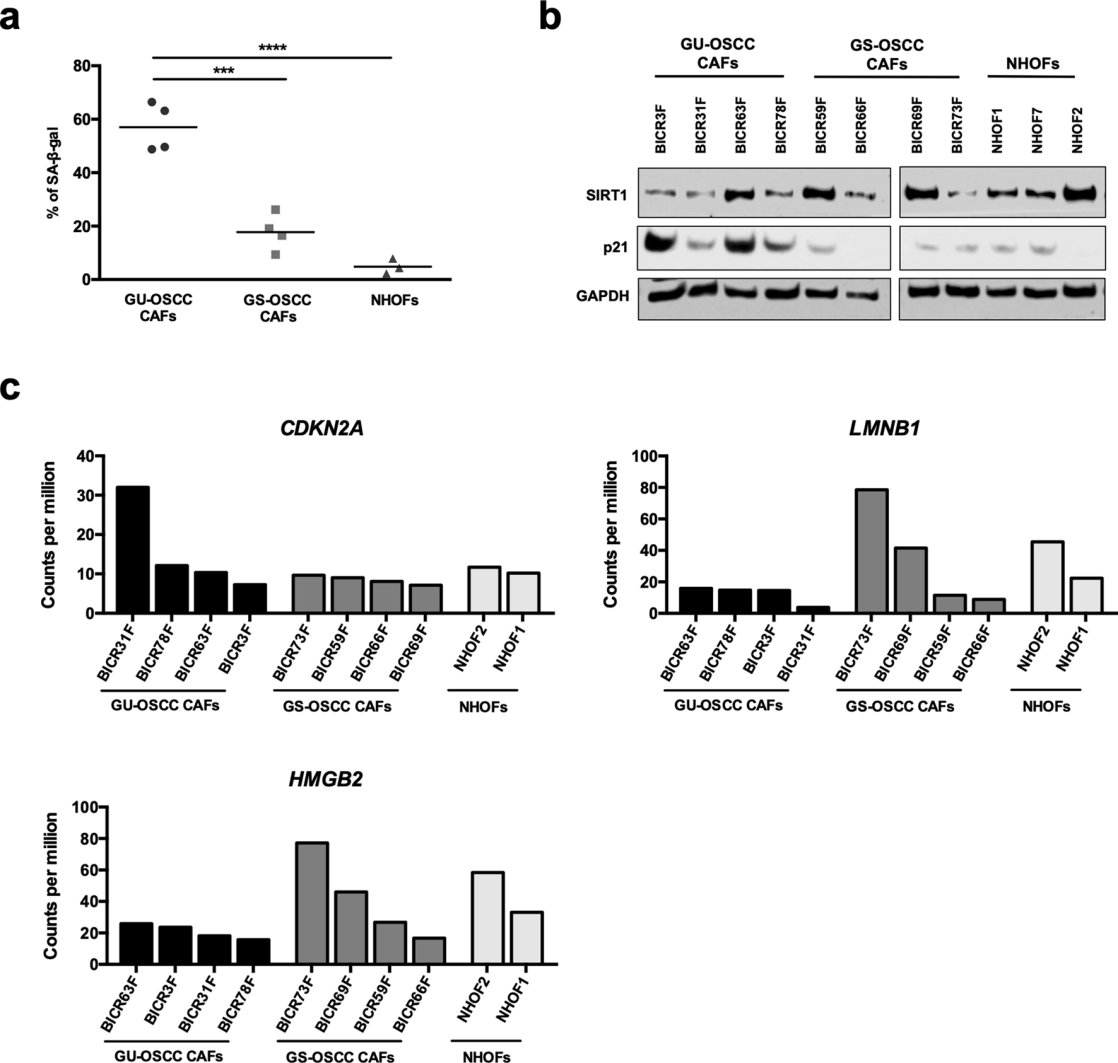
CAFs from GU-OSCCs are highly senescent. (**a**) Scatter dot plot showing the percentage of SA-β-gal positive cells in fibroblast cultures from GU-OSCCs (n = 4), GS-OSCCs (n = 4) and normal oral mucosa (n = 3). The percentage of positive cells in CAFs from GU-OSCCs was significantly higher than both CAFs from GS-OSCCs and normal fibroblasts. ***p < 0.001, ****p < 0.0001 (ANOVA). Representative images of SA-β-gal-stained fibroblasts are shown in Supplementary Fig. S1. (**b**) The loss of SIRT1 is a marker for late senescence and its expression level was generally reduced in CAFs from GU-OSCCs. The expression of p21^Waf1/Cip1^ protein was elevated in CAFs from GU-OSCCs. Representative Western blot images of three independent experiments are presented. Full-length blots are presented in Supplementary Fig. S8. (**c**) Transcript quantification of markers of senescence in CAFs and NHOFs by RNAseq. The transcript abundance of *CDKN2A*, *LMNB1* and *HMGB2* mRNAs are expressed in counts per million.

To investigate the relationship between fibroblast senescence and autophagy, we first quantified the degree of senescence and the number of autophagosomes in a panel of fibroblast strains, including CAFs from GU-OSCCs, CAFs from GS-OSCCs and normal human oral fibroblasts (NHOFs). To measure the degree of senescence, we measured senescence-associated β-galactosidase (SA-β-gal) activity, which is the most commonly used marker to indicate senescence in cultured cells^25^. Previous studies^16,26^ have reported that CAFs from GU-OSCCs are generally more senescent than CAFs from GS-OSCCs and NHOFs, using a variety of markers including SA-β-gal, 8-hydroxy-2-deoxyguanosine (8-oxo-dG) and p16^Ink4a^. Similarly, in the present study CAFs from GU-OSCCs contained significantly more SA-β-gal positive cells compared to CAFs from GS-OSCCs and NHOFs (Fig. 1a; p < 0.001 and p < 0.001, respectively; Supplementary Fig. S1). We also examined the expression of SIRT1, which is down-regulated in senescent cells^27,28^ and reduced levels are considered to be a more specific marker of late senescence^29,30^. Consistent with the level of SA-β-gal activity, Western blot analyses demonstrated that the expression level of SIRT1 was generally lower in senescent CAFs, whilst the levels of the cyclin-dependent kinase inhibitor, p21^Waf1/Cip1^, were higher (Fig. 1b). Analysis of our previously published RNAseq data^31^ showed that other markers of senescence such as reduced levels of *LMNB1*^32^ and *HMGB2*^28,33^ mRNAs were also consistently lower in GU-OSCC CAFs and some of the GS-OSCC CAFs than NHOFs, although this did not reach significance (Fig. 1c). *CDKN2A*/ p16^Ink4a^ mRNA was only high in BICR31 CAFs (Fig. 1c) and this is consistent with the lower levels of p21^Waf1/Cip1^ levels in these cells. In classical senescence, p21^Waf1/Cip1^ levels decline in late senescence and *CDKN2A*/ p16^Ink4a^ levels increase^34,35^ and so BICR31F appears to be at a more advanced stage of senescence. It is acknowledged that CAFs are likely to change in vitro over time and that they are likely to contain different fractions of early and late senescent cells. However, collectively our data show that cultures of CAFs from GU-OSCCs contain a higher proportion of senescent cells than CAFs from GS-OSCCs and NHOFs.

**Figure 2:**
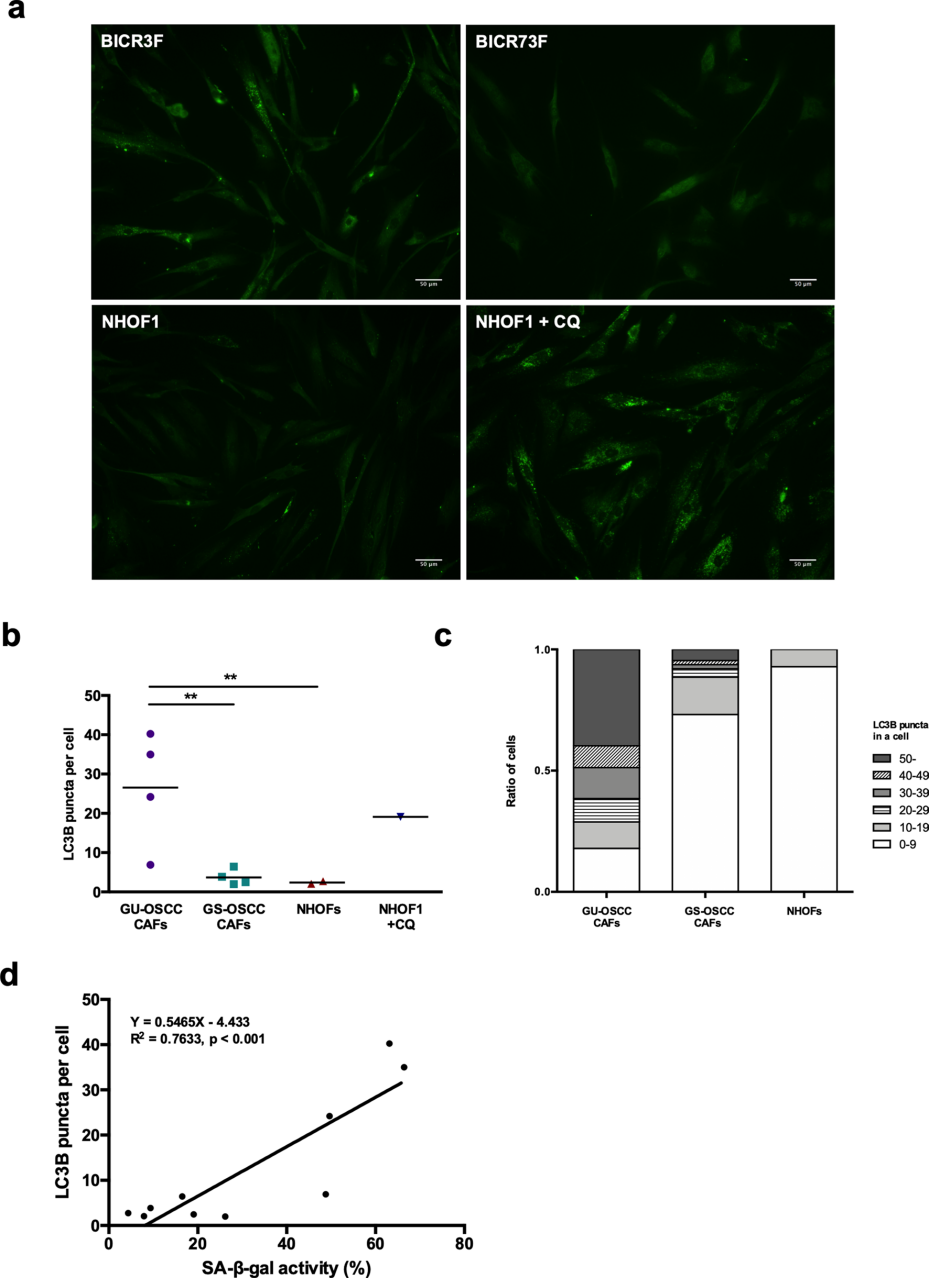
Autophagy and senescence are closely related phenotypes in fibroblasts. (**a**) Representative images of LC3B puncta in immunofluorescent-stained GU-OSCC CAFs (BICR3F), GS-OSCC CAFs (BICR73F) and NHOFs (NHOF1). NHOF1 treated with CQ were used as positive control. Scale bar indicates 50 μm. Additional images of CAFs from GU-OSCCs, GS-OSCCs and NHOFs are shown in Supplementary Fig. S2. (**b**) Quantification of the number of LC3B puncta in fibroblast cultures from GU-OSCCs (n = 4), GS-OSCCs (n = 4) and normal oral mucosa (n = 2), as determined by immunofluorescence staining. A minimum of 350 cells for each fibroblast strain were examined and the number of puncta per cell was quantified using ImageJ software. The number of LC3B puncta was significantly greater (p < 0.01) in CAFs from GU-OSCCs as compared with GS-OSCCs and NHOFs. (**c**) The number of LC3B puncta in each stained cell was counted and the proportion of cells containing different numbers of LC3B puncta is presented in a stacked bar chart. **p < 0.01 (Wilcoxon Mann–Whitney test). (**d**) Positive correlation of the number of LC3B puncta and cells staining positive for SA-β-gal activity in fibroblasts strains (n = 10, Pearson’s r = 0.8737, p < 0.001).

Autophagy is commonly characterised by the redistribution of the LC3B proteins into cytoplasmic puncta, as assessed by fluorescent immunostaining^36^ and this was used in the present study to quantify autophagosomes. Punctate LC3B staining was significantly greater in CAFs from GU-OSCCs compared to GS-OSCCs and NHOFs (Fig. 2a,b; p < 0.001, Supplementary Fig. S2). The proportion of cells with different numbers of LC3B puncta was also calculated. In general, CAFs from GU-OSCCs contained a higher fraction of autophagic fibroblasts than CAFs from GS-OSCC and normal fibroblasts (Fig. 2c). The percentage of cells staining positive for SA-β-gal in all fibroblasts (NHOFs and CAFs) significantly correlated with the number of LC3B puncta (Fig. 2d; Pearson’s r = 0.8737, p < 0.001). This positive correlation suggests that the senescent and autophagic phenotypes in oral fibroblasts are closely related.

### Autophagy is disrupted in CAFs from GU-OSCCs

**Figure 3:**
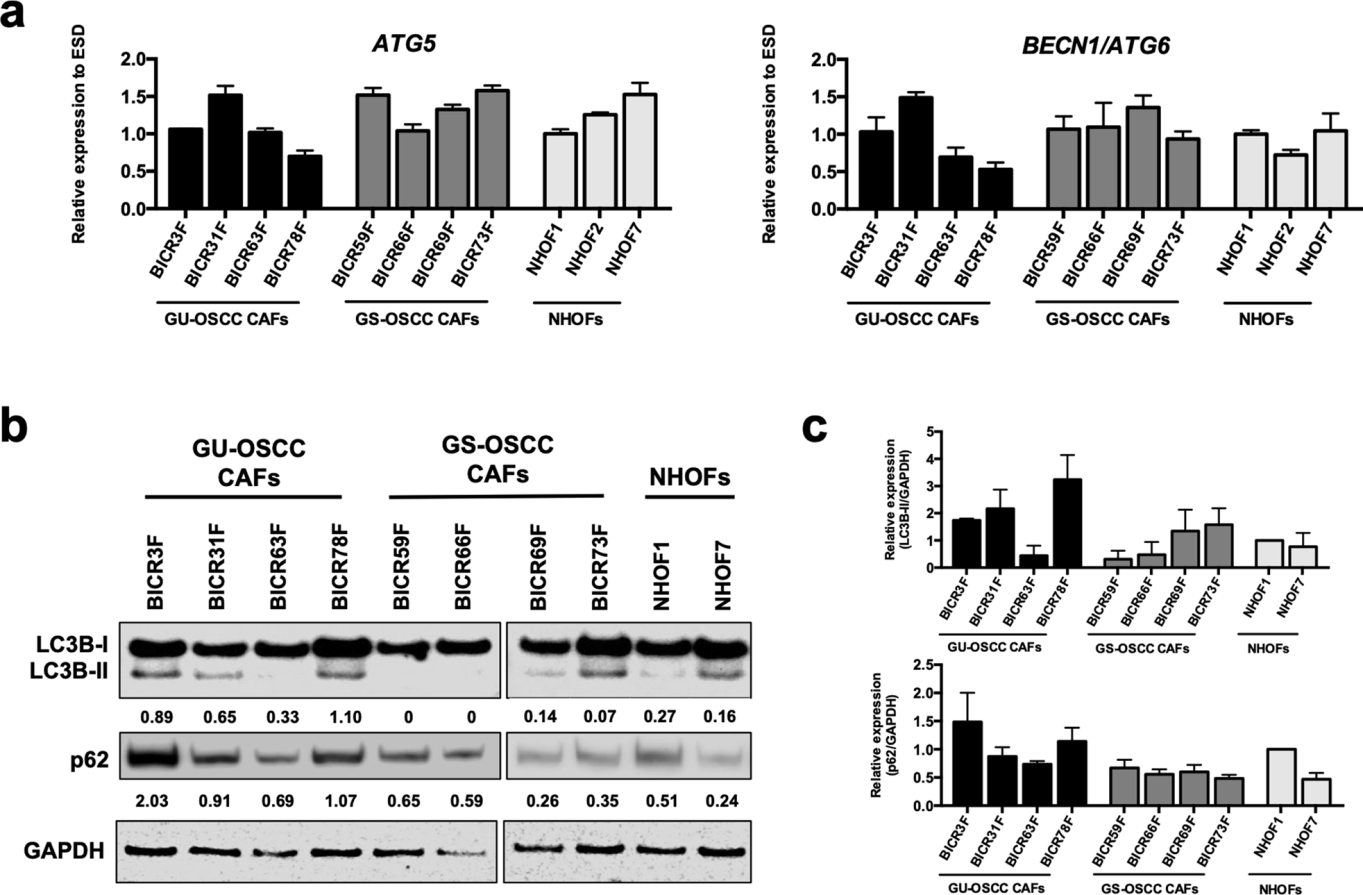
Autophagy is disrupted in CAFs from GU-OSCCs. (**a**) The expression of both *ATG5* and *BECN1/ATG6* mRNAs between all three groups of fibroblasts was not statistically different (ANOVA). The expression of *ATG5* and *BECN1/ATG6* was normalised to *ESD* and the data were expressed as mean ± SD of triplicates. The data presented are representative of two independent experiments. The expression of a number of *ATG* genes derived by RNAseq experiment is presented in Supplementary Fig. S3. (**b**) Expression of LC3B-II and p62 protein in CAFs and NHOFs, as determined by Western blotting. The images shown are representative of three independent experiments. Full-length blots are presented in Supplementary Fig. S8. (**c**) Mean densitometric values from three experiments were normalised to GAPDH and expressed relative to NHOF1 (= 1).

The elevation in the number of autophagosomes in CAFs may represent either a true upregulation of autophagic degradation (autophagic flux) or an inhibition in the completion of the autophagic pathway (decrease in autophagic degradation). Hence, the sole determination of the number of autophagosomes is not sufficient to determine overall autophagic activity. To distinguish between these two physiologically different scenarios, the expression of a number of *ATG* genes was analysed using our previously published RNAseq data^31^. We selected *ATG5*, *BECN1/ATG6*, *ATG7*, *ATG10*, *ATG12*, *ATG13*, *ATG14* and *ATG16L1* because they are widely studies upstream regulators of autophagy^37^. There were no statistically significant differences in the expression of these *ATG* genes between CAFs from GU-OSCCs, CAFs from GS-OSCCs and NHOFs (Supplementary Fig. S3). The results were validated with qPCR for *ATG5*, which is required during autophagosomal elongation and *BECN1/ATG6* that regulates the initiation of autophagosome formation (Fig. 3a). These results imply that the accumulation of autophagosomes observed in CAFs from GU-OSCCs was unlikely to be related to the induction of autophagic flux.

The cytoplasmic protein, p62, is an autophagic substrate that binds to LC3 selectively and p62 itself is degraded efficiently by autophagy. Therefore, changes in the protein level of p62 can be used to indicate a defect in the autophagic pathway or the turnover of protein aggregates during late autophagy and p62 expression inversely correlates with autophagic flux^36^. There was a concomitant accumulation of p62 with LC3B-II protein in fibroblasts from GU-OSCCs, and the expression of these proteins was generally higher in these cells relative to GS-OSCCs and NHOFs (Fig. 3b,c). It is noteworthy that whilst there was heterogeneity in the expression of p62 with LC3B-II, the lowest levels of expression amongst the GU-OSCC CAFs was observed in BICR63F, which is the least senescent of the GU-OSCC CAF strains (Fig. 1b; Supplementary Fig. S1b). These results suggested that there was impairment in the later stages of the autophagic pathway in GU-OSCC CAFs fibroblasts. Lysosomal protease inhibitors, such as chloroquine (CQ), can be used to determine whether autophagosome accumulation is caused by increased autophagic flux or impaired autophagic degradation^38^. The slow growing, highly senescent nature of the GU-OSCC CAFs and the toxic nature of CQ meant that this was not possible in the present study. However, limited experiments using the least senescent GU-OSCC CAF, BICR63F, together with BICR73F (GS-OSCC) and NHOFs, indicated that LC3-II levels did not accumulate in CQ-treated BICR63F cells, an effect that was evident in BICR73F and NHOFs (Supplementary Fig. S4). This result further supports the notion that there is autophagic impairment in CAFs from GU-OSCCs. Collectively, these data suggest that the accumulation of autophagosomes in senescent CAFs from GU-OSCCs was likely due to disruption at the degradation stage of the autophagic pathway.

### Inhibition of basal autophagy in normal fibroblasts induces activation and senescence

**Figure 4:**
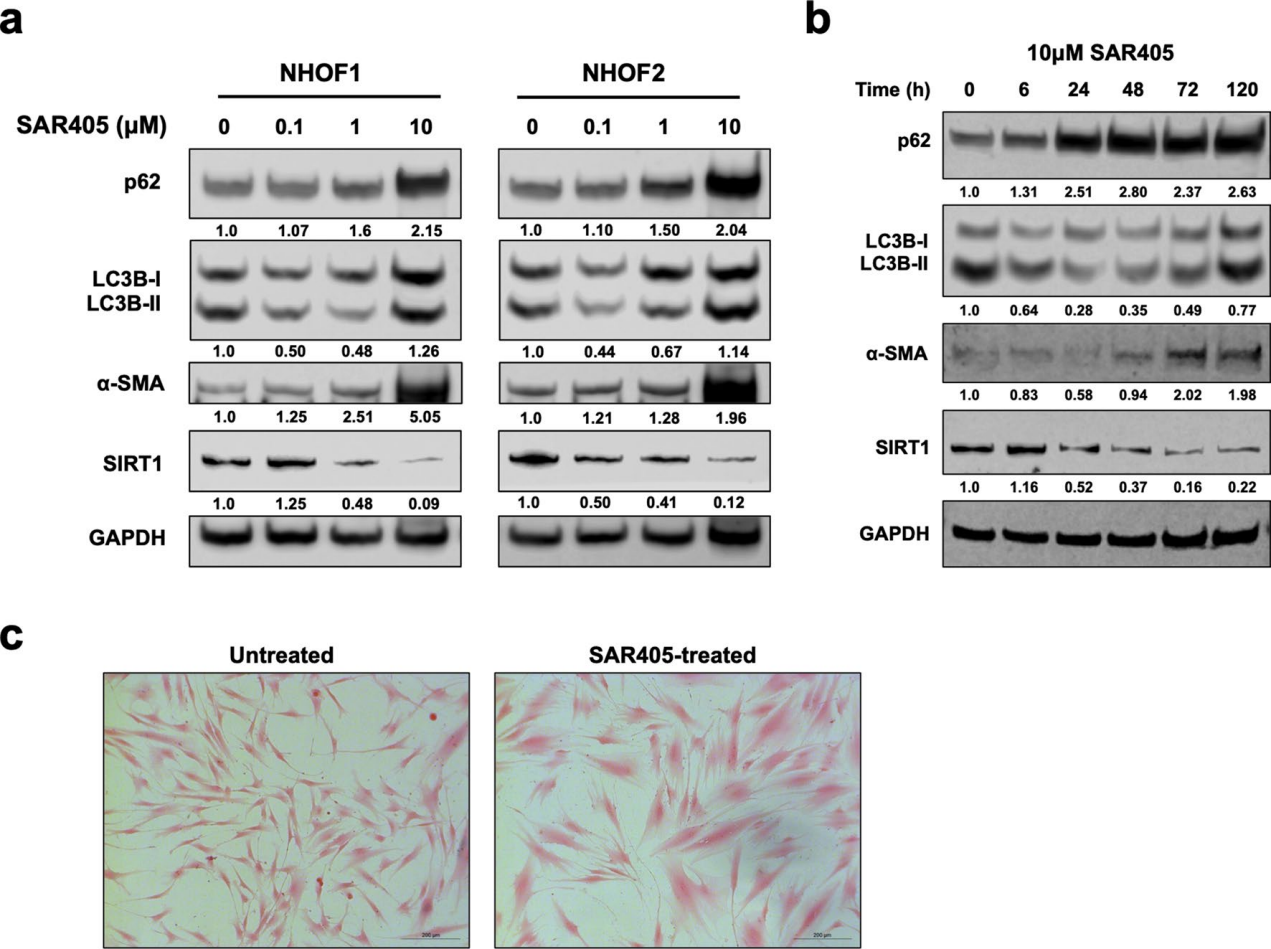
Inhibition of autophagy in normal fibroblasts induces activation and senescence. (**a**) Treatment of normal oral fibroblasts (NHOF1 and NHOF2) with increasing doses of the specific Vps34 inhibitor, SAR405 for 72 h. (**b**) Treatment of NHOF2 with 10 μM SAR405 for up to 120 h. Autophagy inhibition with SAR405 was shown to induce fibroblast activation (induction of α-SMA) and senescence (loss of SIRT1) by Western blotting. GAPDH was used as a loading control. Densitometric values were normalised to GAPDH and expressed relative to fibroblasts treated with vehicle control (= 1). Full-length blots are presented in Supplementary Fig. S8. (**c**) Normal oral fibroblasts displayed cell morphology typical of senescent cells after SAR405 (10 μM, 120 h) treatment. Scale bar indicates 200 μm.

It was previously shown that fibroblast activation and senescence were closely related in a similar panel of OSCC-derived CAFs^16^ and the present study has demonstrated a close association between autophagy and senescence. Therefore, we examined fibroblast activation and senescence in NHOFs after inhibiting basal autophagy. Normal fibroblasts were treated with SAR405, a specific autophagic inhibitor that selectively targets vascular protein sorting 34 (Vps34, also known as PI3K class III)^39^, which is commonly found in protein complexes with BECN11/ATG6 that is required for autophagy initiation. Treatment of normal fibroblasts with increasing doses of SAR405 (1–10 μM) for 72 h resulted in a dose-dependent accumulation of p62 protein and levels of LC3B-II, demonstrating that there was a dose-dependent inhibition of autophagy (Fig. 4a; Supplementary Fig. S5). In addition, upregulation of α-SMA and downregulation of SIRT1 were also observed in a dose-dependent manner following SAR405 treatment (Fig. 4a). As the most prominent effect was observed with 10 μM SAR405, this dose was used to treat NHOF2 cells for up to 120 h to inhibit autophagy. SAR405 inhibited autophagy as indicated by an increase in p62 levels and LC3B-II) from 24 h onwards (Fig. 4b). Treatment of cells with SAR405 resulted in an increase in α-SMA expression after 72 h, with a gradual decrease in SIRT1 expression from 24 to 120 h, which was accompanied by the development of a flatter cell morphology and enlarged cell size, consistent with senescence (Fig. 4c). The results demonstrate that inhibition of autophagy with SAR405 induced both activation and senescence in normal oral fibroblasts, suggesting these two biological responses are regulated by autophagy.

### TGF-β1 induces autophagic flux, activation and senescence in normal fibroblasts

**Figure 5:**
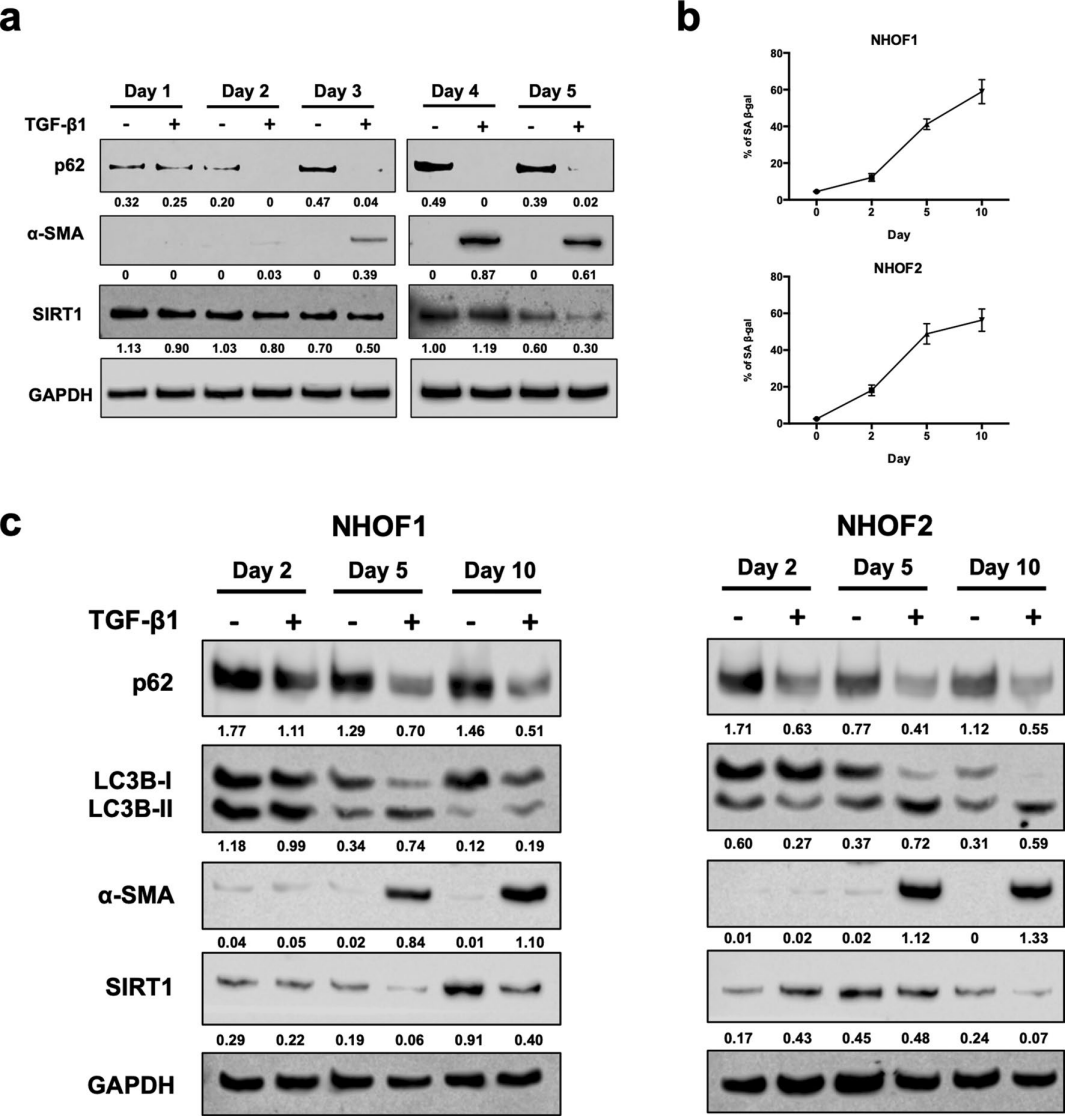
TGF-β1 induces autophagic flux, activation and senescence in normal fibroblasts. (**a**) Induction of autophagic flux, α-SMA expression and SIRT1 downregulation following treatment of NHOF1 with 4 ng/mL TGF-β1 for 5 days. Densitometric values are normalised to GAPDH. (**b**) Progressive increase of SA-β-gal activity in NHOF1 and NHOF2 following TGF-β1 treatment for 10 days. (**c**) Western blotting for autophagic (p62 and LC3B-II), activation (α-SMA) and senescent (SIRT1) markers following treatment of normal fibroblasts (NHOF1 and NHOF2) with 4 ng/mL TGF-β1 for 10 days. TGF-β1 induced autophagic flux, activation and senescence with autophagic flux preceding activation, followed by senescence in normal fibroblasts. The densitometric data are expressed as relative expression normalised to GAPDH. The Western blots are representative of three independent experiments. Full-length blots are presented in Supplementary Fig. S8.

GU-OSCC CAFs have been reported to overexpress TGF-β1 and TGF-β2^40^ and that GU-OSCCs induce senescence in oral fibroblasts in a TGF-β-dependent manner^16^. Specifically, TGF-β1 has been shown to induce both activation and senescence in normal oral fibroblasts^16,17^. Therefore, we examined whether autophagy was altered during induction of fibroblast activation and senescence in response to TGF-β1. NHOF1 cells were first treated with 4 ng/mL of TGF-β1 for 5 days. The results indicated that TGF-β1 induced autophagic flux as demonstrated by a reduction in expression of p62 (Fig. 5a), an effect that was apparent in the presence of chloroquine which inhibits autophagic flux by decreasing autophagosome-lysosome fusion (Supplementary Fig. S5). Further, TGF-β1 induced fibroblast activation, as indicated by the elevation of α-SMA expression from day 3 and down-regulated SIRT1 after 5 days, suggesting that senescence was being induced (Fig. 5a). Therefore, to further investigate the relationship between autophagic flux and late senescence, NHOF1 and NHOF2 were treated with 4 ng/mL TGF-β1 for 10 days and this resulted in a progressive rise in SA-β-gal activity (Fig. 5b; Supplementary Fig. S6), indicating that senescence was induced in these cells. Late senescence was confirmed with the loss of SIRT1 protein and fibroblast activation was indicated by the up-regulated expression of α-SMA (Fig. 5c). Autophagic flux was induced as demonstrated by an increase in the expression of LC3B-II protein together with the downregulation of p62 expression following TGF-β1 treatment and this effect was apparent from day 2 onwards (Fig. 5c). Taken together, these results demonstrate that TGF-β1 induced autophagic flux, activation and senescence in NHOFs, with autophagic flux preceding activation, followed by senescence.

### Autophagic impairment and autophagic flux induces activation and senescence in normal fibroblasts

**Figure 6:**
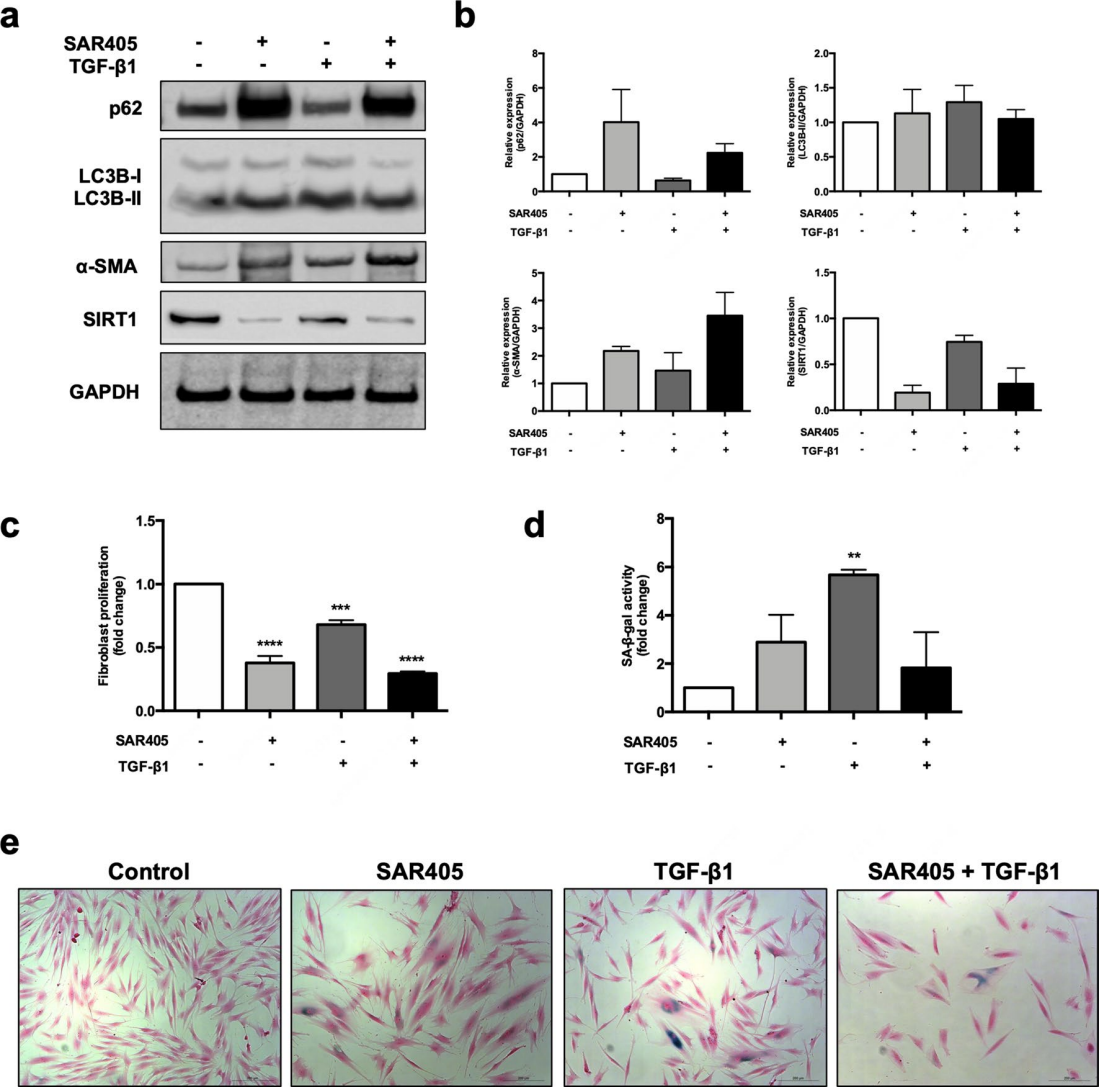
Autophagic impairment and autophagic flux induce activation and senescence in normal oral fibroblasts. (**a**) Treatment of NHOF2 with SAR405 for 120 h inhibited autophagy (increased LC3B-II and p62), while TGF-β1 induced autophagic flux (high LC3B-II and low p62). Both SAR405 and TGF-β1 induced the expression of α-SMA and down-regulated SIRT1 levels in NHOF2. Full-length blots are presented in Supplementary Fig. S8. (**b**) Mean densitometric values from three experiments were normalised to GAPDH and expressed relative to fibroblasts treated with vehicle control (= 1). (**c**) The proliferation of normal fibroblasts was significantly lower after SAR405 or TGF-β1 treatment as compared to control (= 1). Data are presented as mean ± SEM. ***p < 0.001, ****0.0001, ANOVA compared to control. (**d**) Fibroblasts with autophagic inhibition (SAR405) and autophagic activation (TGF-β1) displayed a senescent phenotype of higher SA-β-gal positivity. Data are presented as mean ± SEM. **p < 0.01, ANOVA compared to control. (**e**) Representative images of SA-β-gal staining of SAR405- or/and TGF-β1-treated NHOF2. Treated fibroblasts displayed typical features of senescent cells with enlarged cell size, flat-shaped and contractile myofibroblast-like phenotypes. Scale bar indicates 200 μm.

As autophagy was induced prior to activation and senescence following the treatment of normal fibroblasts with TGF-β1, we next investigated whether the inhibition of autophagy would block TGF-β1-induced fibroblast activation and senescence. To do this, normal oral fibroblasts (NHOF2) were treated for 120 h with 10 μM SAR405 to inhibit autophagy in the absence or presence of TGF-β1 (4 ng/mL). Autophagy inhibition was confirmed by high LC3B-II and high p62 expression, whereas autophagic flux was confirmed by increased LC3B-II and reduced p62 levels (Fig. 6a,b; Supplementary Fig. S5). Both the inhibition of autophagy with SAR405 and the induction of autophagic flux with TGF-β1 increased the expression of α-SMA, in addition to suppressing SIRT1 expression in fibroblasts (Fig. 6a,b). Treatment of cells with SAR405 and TGF-β1 inhibited cell proliferation (Fig. 6c), which is consistent with the growth characteristics of senescent cells. In line with these observations, SA-β-gal staining showed that treatment of fibroblasts with SAR405 or TGF-β1 resulted in an increase in the number of SA-β-gal positive cells (SAR405 = 2.89 ± 1.13 fold, p = 0.31; TGF-β1 = 5.67 ± 0.21 fold, p < 0.01) compared to untreated controls (= 1; Fig. 6d,e), although the increase was not statistically significant for cells treated with SAR405. Autophagic flux induced by TGF-β1 was abrogated when the cells were co-treated with SAR405 and there was clear evidence of fibroblast activation in these conditions (Fig. 6a,b). The effects of SAR405 on TGF-β1-induced senescence were less clear because whilst SIRT1 levels and cell proliferation were reduced, SA-β-gal staining was also reduced (Fig. 6d,e). Collectively, these results confirm that both activation and senescence were induced in normal oral fibroblasts following autophagy inhibition with SAR405 and the induction of autophagic flux with TGF-β1. Further, the data demonstrate that SAR405 antagonised the effects of TGF-β1 on the induction of autophagy and that NHOFs became activated following co-treatment of cells with SAR405 and TGF-β1.

### Conditioned media from fibroblasts with altered autophagic activity induces epithelial migration and invasion

**Figure 7:**
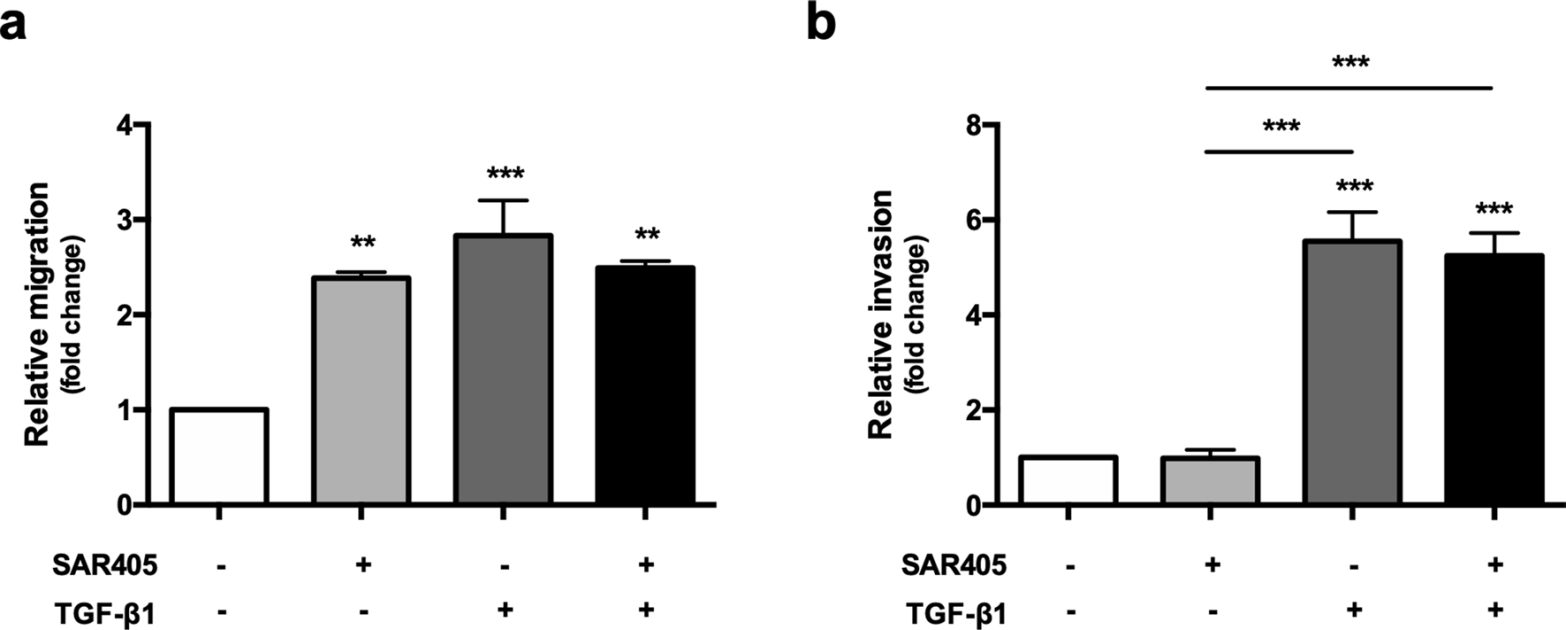
Migration and invasion of OSCC cells following SAR405-induced autophagic inhibition and TGF-β1-induced autophagic flux in normal oral fibroblasts. Transwell migration and invasion assays using H376 OSCC cells were carried in serum-free conditions for 24 and 48 h, respectively. The lower chamber contained either CM from normal oral fibroblasts NHOF2 (control), SAR405-treated NHOF2, TGF-β-treated NHOF2 or combination of SAR405 + TGF-β1-treated NHOF2. The number of migrated (**a**) and invaded (**b**) H376 cells are expressed as a relative to control (= 1). The data presented are representative of three independent experiments. Data are presented as mean ± SD. **p < 0.01, ***p < 0.001 and are shown for ANOVA compared to control unless otherwise indicated.

It has been shown previously that the induction of the activated and senescent phenotypes in NHOFs by TGF-β1 resulted in the secretion of factors that enhanced the migration and invasion of OSCC cells similar to GU-OSCC CAFs^16,26^. As we have shown that fibroblast activation occurred following the treatment of NHOFs with both SAR405 (inhibition of autophagy, Fig. 4) and TGF-β1 (induction of autophagic flux, Figs. 5 and 6), we examined whether fibroblasts activated by these two mechanisms could also affect OSCC cell migration and invasion. Conditioned media (CM) were collected from NHOFs after 5 days of autophagy inhibition (SAR405-treated) or autophagic-activation (TGF-β1-treated). CM from SAR405- or TGF-β1-treated NHOF2 significantly increased the migration of H376 cells (SAR405 p < 0.01, TGF-β1 p < 0.001; Fig. 7a) relative to CM from untreated cells. However, no additional effect on the migration of H376 cells was observed in the presence of CM from NHOF2 cells treated with both SAR405 and TGF-β1, suggesting that SAR405-induced autophagy inhibition and TGF-β1-induced autophagic flux might influence OSCC cell migration by similar mechanisms. The invasive behaviour of H376 cells was not affected following treatment with CM from SAR405-treated NHOF2 (0.98 ± 0.18 fold) but was significantly enhanced by CM from TGF-β1-treated NHOF2 (5.55 ± 0.61 fold) (TGF-β1; Fig. 7b). In the presence of CM from SAR405 + TGF-β1-treated NHOF2 (5.25 ± 0.48 fold), the invasive effect was comparable to that with TGF-β1 alone.

Collectively, our data indicate that alterations in autophagy (either inhibition with SAR405 or induction of autophagic flux with TGF-β1) which result in fibroblast activation enhanced the migration of OSCC and that additional effects of TGF-β1 are required to stimulate tumour cell invasion.

## Discussion

The phenotypic and functional changes that occur in fibroblasts as a result of cellular stress, such as autophagy, activation and senescence, are likely to mediate many of the pro-tumourigenic properties of CAFs. In the present study, the autophagic phenotypes of CAFs from GU-OSCCs and GS-OSCCs were characterised and these features were compared with normal fibroblasts (NHOFs). It has been shown previously that CAFs from GU-OSCCs, which are associated with genetically unstable and more aggressive tumours, are activated and contain more senescent cells than CAFs from GS-OSCCs and NHOFs^16^. Specifically, GU-OSCC CAFs were shown to express higher levels of α-SMA, a marker of fibroblast activation, together with several markers of senescence, including SA-β-gal, 8-oxo-dG and p16^Ink4a^ and that the activated and senescent phenotypes were closely related^16^. In the present study, we show that the levels of SIRT1, a specific marker of late senescence in fibroblasts^29,30^, were also generally lower in GU-OSCC CAFs compared to CAFs from GS-OSCCs and NHOFs. BICR63F, the GU-OSCC CAF with the highest level of SIRT1, was the least senescent of these cells, which further validates SIRT1 as a marker of senescence in fibroblasts. These in vitro observations are likely to be relevant to the situation in vivo because, whilst CAFs continue to divide in vivo, it is likely that they divide more slowly and senesce more quickly. A high percentage of CAFs in vivo are also p16^Ink4a^ postive and therefore likely to be senescent^17^. There is indeed evidence that CAFs do have a reduced replicative lifespan when compared with their normal counterparts^41^ supporting this hypothesis.

Having confirmed previous reports showing that the majority of CAFs from GU-OSCCs contain a higher proportion of senescent cells^16,26^, we next examined autophagy in CAFs and NHOFs. Autophagy is activated in a response to damaged telomeres and organelles such as mitochondria and it is likely that senescence is triggered only when autophagy is disrupted. By quantifying LC3B puncta as a marker to assess autophagosome numbers, we showed that senescent CAFs from GU-OSCCs also displayed autophagosome accumulation, results that are in agreement with a previous report showing increased autophagosome numbers in CAFs from head and neck cancer^42^. We further showed that autophagosome accumulation, as indicated by the number of LC3B puncta, directly correlates with the degree of senescence, indicating that autophagy and senescence are closely associated in fibroblasts. This association is supported by the increase lysosomal mass in senescent cells and the function of β-galactosidase as a lysosomal enzyme^21,43,44^. The accumulation of autophagosomes in CAFs from GU-OSCCs was most likely due to autophagic impairment because there was a general increase in p62 and LC3B-II protein levels in these cells without any obvious alterations in *ATG* gene expressions, indicating disruption at the degradation stage of the autophagic pathway^36^. This was supported by preliminary observations that LC3-II levels accumulated in BICR73F (GS-OSCC CAF) and NHOFs cells treated with CQ, but this was not observed in CQ-treated BICR63F cells (the least senescent GU-OSCC CAF). Confirmatory studies in highly senescent GU-OSCC CAFs were not possible because of the very slow proliferation rate of these cells and the toxic nature of CQ. In summary, we show that in general CAFs from GU-OSCCs concomitantly express markers of autophagy and senescence and that these processes are closely linked in fibroblasts. We also we present preliminary evidence to indicate that autophagosome accumulation in senescent CAFs is due to autophagic impairment.

As our data showed that autophagy and senescence are closely related in fibroblasts and that autophagy is likely impaired in senescent CAFs, we examined activation and senescence in normal fibroblasts following the inhibition of autophagy with SAR405, which has been shown to be a selective and potent inhibitor of autophagy^39^ that is widely used^45,46^. We demonstrate that inhibition of basal autophagy with SAR405 in NHOFs induces both fibroblast activation and senescence, which highlights the homeostatic role of autophagy in regulating these processes in normal fibroblasts. There is increasing evidence to show that autophagy is required for CAF activation^47^ and indeed, CAFs from head and neck cancers have an increased rate of basal autophagy with increased numbers of autophagosomes, although the degree of senescence in these cultures was not reported^42^. Our data show that inhibition of autophagy can also result in fibroblast activation and senescence, indicating that a variety of stimuli that affect basal autophagy could result in the induction of CAF phenotypes. TGF-β1 has been shown to induce both activation and senescence in normal oral fibroblasts^16,17^ and this is likely to be relevant physiologically because GU-OSCCs induce senescence in NHOFs in a TGF-β-dependent manner^16^ and GU-OSCC CAFs overexpress both TGF-β1 and TGF-β2^35^. In the present study, we have extended these observations and demonstrate that TGF-β1 induced autophagic flux and this occurred prior to the development of fibroblast activation and senescence. Therefore, both the inhibition of autophagy with SAR405 and the stimulation of autophagy with TGF-β1 resulted in activation and senescence in normal oral fibroblasts. These observations are most likely to be explained by the highly context-dependent nature of autophagy, as seemingly paradoxical observations have been reported previously in other cell systems. For example, autophagy has been reported to be a pre-requisite for senescence^23^ and paradoxically also inhibits ageing^48^ and the removal of senescent cell inhibits ageing in mouse models^49^. Together, our results demonstrate that deregulated autophagy induced fibroblast activation and senescence and, in response to TGF-β1, autophagy was induced prior to the development of the activated and senescent phenotypes in normal oral fibroblasts.

In this study, we have investigated how autophagy influenced the development CAF phenotypes, such as fibroblast activation and senescence and we show that both the inhibition of autophagy with SAR405 and the stimulation of autophagy with TGF-β1 induce these processes. We also show that SAR405 abrogated the autophagic flux normally induced by TGF-β1 but the cells remained were still activated, as shown by the increased expression of α-SMA (Fig. 6a). This latter result might be expected as SAR405 functions to inhibit the initiation step of the autophagic process^39^ and our data showed that SAR405 and TGF-β1 increased α-SMA expression in NHOFs. We next wanted to begin to investigate the biological significance of deregulated autophagy in fibroblasts. To do this, we used CM collected from NHOFs under conditions of autophagic flux (TGF-β1-induced) and autophagy inhibition (SAR405-treated) and examined the effect on OSCC cell migration and invasion in vitro. Our results showed that both treatment of NHOFs with SAR405 to inhibit autophagy or TGF-β1 to induce autophagic flux resulted in the secretion of factors that enhanced the migration OSCC cells in transwell assays. These apparently contradictory observations are likely explained by the fact that SAR405 and TGF-β1 or co-treatment with both agents promoted fibroblast activation (and possibly senescence) in NHOFs. It has been reported previously, for example, that activated fibroblasts can promote invasion even before senescence is evident^16^ and non-senescent CAFs are still associated with OSCC progression^17^. We also showed that CM from TGF-β1-treated but not SAR405-treated cells promoted the invasion of OSCC cells through Matrigel and that SAR405 did not alter the effects of TGF-β1, suggesting that the effects of SAR405 and TGF-β1 are distinct. Taken together, these data show that fibroblast activation and/or senescence following deregulated autophagy is sufficient to enhance migration but not invasion and suggest that TGF-β1 had additional effects that are independent of activation/senescence to promote invasion. In this regard, it is interesting to note that fibroblasts induced to senesce with TGF-β1 are not only α-SMA-positive, but also secrete numerous soluble factors that promote tumour invasion, such as MMP-2^17^. It is also important to also recognise that the induction of autophagy in fibroblasts results in the secretion of pro-tumorigenic factors^21,42,50^ that may be independent from activation and/or senescence.

Our observations that autophagy impairment was associated with senescent CAFs from GU-OSCCs, whilst autophagy was enhanced in normal fibroblasts induced to senesce by TGF-β1, a cytokine that has been shown to be up-regulated in senescent CAFs from GU-OSCCs, appear paradoxical. However, the precise role of autophagy in regulating cellular senescence is thought to be complex and context dependent. For example, it has been shown that autophagic flux is activated in senescent cells and that senescence may be secondary to autophagic induction^21,51^. Conversely, a concept known as “cellular senescence with autophagy” can be perceived as a failure of the homeostatic role of basal autophagy in attempting to suppress senescence due to the accumulation of potentially harmful entities (e.g. damaged proteins or increased mitochondrial dysfunction)^51,52^. Several studies have highlighted the critical role of basal autophagy in maintaining homeostasis and preventing cellular senescence. For example, García-Prat et al.^53^ demonstrated that defective autophagy triggers senescence in muscle stem cells and autophagy maintains stemness of cells by suppressing senescence. Therefore, it is possible that fibroblast senescence could be stimulated via the up-regulation of TGF-β by OSCC-derived ROS, as suggested by Hassona et al.^16^ which would increase autophagic flux and this is subsequently followed by a breakdown of basal autophagy leading to the accumulation of autophagosomes and maintenance of senescence in CAFs.

In summary, we show that CAFs from GU-OSCCs concomitantly express markers of senescence and contain increased numbers of autophagosomes, indicating that the processes of senescence and autophagy are closely related. Further, we show that deregulation of autophagy in normal fibroblasts, either by inhibition with SAR405 or activation with TGF-β1, induces fibroblast activation and senescence: in response to TGF-β1, autophagy is induced prior to the development of the activated and senescent phenotypes. Lastly, we show that both SAR405- and TGF-β1-treated NHOFs enhance OSCC cell migration but only TGF-β1-treated cells increase OSCC invasion through Matrigel, indicating that TGF-β1 has additional effects that are independent of fibroblast activation/senescence. The results highlight a key role for autophagy in the development of myofibroblast and CAF phenotypes.

## Materials and methods

### Reagents

Recombinant human TGF-β1 was obtained from Miltenyi Biotec (Bergisch Gladbach, North Rhine-Westphalia, Germany) and SAR405 from Calbiochem (Merck Millipore, Burlington, MA, USA).

### Cell strains and lines

A series of fibroblast strains consisting of NHOFs (NHOF1, NHOF2, NHOF7), CAFs from GS-OSCCs (BICR59F, BICR66F, BICR69F, BICR73F) and CAFs from GU-OSCCs (BICR3F, BICR31F, BICR63F, BICR78F) were used in this study. The derivation, molecular characterisation and culture of these fibroblast strains has been published previously^26^. A well-characterised human malignant oral keratinocyte cell line, H376, was also used and has been described previously^54^.

### SA-β-gal staining

SA-β-gal activity was measured histochemically using a senescence detection kit (BioVision, Milpitas, CA, USA), according to the manufacturer’s protocol. Positively blue-stained senescent cells were counted and the percentage of positive cells was calculated (relative to the total number of cells). A minimum of 100 cells was counted for each sample and time-point.

### Immunofluorescence staining

4.5–5 × 10^4^ cells were seeded onto a Nunc Lab-Tek II Chamber Slide (Thermo Scientific, Waltham, MA, USA) and allowed to adhere overnight at 37 °C. The following day, cells were fixed with ice-cold methanol: acetone (1:1) for 10–15 min. The fixed cells were rinsed three times with washing buffer (0.1% Tween-20 in 1X PBS) and non-specific binding site was blocked with blocking buffer (10% goat serum diluted in washing buffer) for 1 h at room temperature. The cells were incubated for 2 h with anti-LC3B antibody (ab51520, Abcam, Cambridge, UK; 1:1000) diluted in blocking buffer. Following three 10 min washes in washing buffer, the slides were incubated with AlexaFluor 488-conjugated goat anti-rabbit secondary antibody (A11034, Thermo Fisher Scientific, Waltham, MA, USA; 1:1000) diluted in blocking buffer for 1 h in room temperature. The slides were further washed in washing buffer three times, 10 min each in the dark and mounted with Vectashield antifade mounting medium with DAPI (Vector Laboratories, Burlingame, CA, USA). The slides were observed under a confocal microscope (LSM 510 META, Zeiss, Oberkochen, Germany) and the staining analysed using ImageJ software^55^.

### Reverse transcription-quantitative PCR (RT-qPCR)

Total RNA was extracted from cell pellets using an RNeasy Mini kit (Qiagen, Hilden, Germany) and subjected to reverse transcription using High-Capacity cDNA Reverse Transcription kit (Applied Biosystems, Foster City, CA, USA) according to the manufacturers’ protocols. qPCR was performed in triplicate using an ABI 7500 Fast Real-Time PCR System (Applied Biosystems, Foster City, CA, USA) with FastStart Universal Probe Master (Rox; Roche Molecular Diagnostics, Rotkreuz, Switzerland). Commercially available TaqMan Gene Expression Assays for *ATG5* (Hs00355492_ml), *BECN11/ATG6* (Hs00186838_ml) and *ESD* (Hs00382667_ml) were purchased from Applied Biosystems, USA. The qPCR results were analysed using 7500 Software v2.0 (Applied Biosystems, USA) and fold changes in gene expression were determined using the comparative threshold cycle method (ΔΔCt). The target gene expression was normalised against *ESD* expression as an endogenous control.

### Western blotting

Cells were lysed with ice-cold RIPA lysis buffer (Bio Basic, Markham, ON, Canada) containing freshly added phosphatase (halt phosphatase inhibitor; Thermo Scientific, Waltham, MA, USA) and protease inhibitors (cocktail set III, Calbiochem, Merck Millipore, Burlington, MA, USA). Western blotting was carried out using standard protocols. Antibodies against α-SMA and p21^Waf1/Cip1^ were purchased from Dako (Santa Clara, CA, USA; M0851) and Merck (Boston, MA, USA; clone CP74, 05-655) respectively, whilst antibodies against LC3B (ab51520), p62 (ab56416), SIRT1 (ab32441) and GAPDH (ab9485) were obtained from Abcam (Cambridge, UK). These antibodies have been widely used and characterised and were shown to be specific in Western blots (Supplementary Fig. S7). Bound antibodies were detected with WesternBright Sirius enhanced chemiluminescence (ECL) reagent (Advansta, San Jose, CA, USA) using an Odyssey FC Imaging System (LI-COR Biosciences, Lincoln, NE, USA). Densitometric analyses were conducted using ImageJ software^55^.

### Collection of conditioned media

NHOFs were cultured as normal in the presence of 10 μM SAR405 or 4 ng/mL TGF-β1 for 5 days (with media changed every 48 h). The cells were washed twice with PBS and serum-free media, and then further incubated with serum-free medium for 48 h. The conditioned media (CM) were centrifuged at 2000 rpm for 5 min to remove debris and dead cells. The remaining viable cells in the flasks were trypsinised and counted for normalisation. CM were stored at − 80 °C and diluted with fresh supplement-free medium at a 1:1 ratio prior to use.

### Transwell migration and invasion assays

OSCC cell migration and invasion were assessed using Transwell Boyden chambers. Polycarbonate filter inserts (8 μM pore size; Corning, NY, USA) were coated with a layer of 10 μg/mL fibronectin or with 250 μg/mL growth factor-reduced Matrigel (Corning, NY, USA) in medium for migration or invasion assays, respectively. Prior to cell seeding in the upper chambers, OSCC cells were first serum-starved overnight with serum-free medium and treated with 10 μg/mL mitomycin C (Merck Millipore, Burlington, MA, USA) for 2 h at 37 °C to negate any potential effects of proliferation. For migration assays, 5 × 10^5^ OSCC cells in 200 μL medium were seeded into the upper chambers and 500 μL of chemoattractant was loaded into the lower chambers. After incubation for 20 h, the non-migrated cells in the upper compartment were removed by swabbing the inserts with cotton buds. For invasion assays, the upper chamber contained 5 × 10^5^ OSCC cells in 500 μL medium while the lower chamber was filled with 750 μL of chemoattractant and the cells allowed to invade for 48 h. The migrated/invaded cells were stained with 0.1% crystal violet and counted in five random fields at 20X magnification.

### Statistical analyses

GraphPad Prism 6.0 software (GraphPad Software, San Diego, CA, USA) was used for all the statistical analyses. Data are reported as mean ± SD or SEM. Mann–Whitney’s U test was performed to evaluate the statistical difference between different CAF samples. Student *t*-test was used for comparisons between two groups and one-way ANOVA was used for more than two-group comparisons. Pearson’s correlation was performed to study the correlation between autophagic and senescent phenotypes in CAF samples. *p* values < 0.05 were considered as statistically significant.

## Supplementary Information

Supplementary Figures.
