# Author Correction: Autophagy is deregulated in cancer-associated fibroblasts from oral cancer and is stimulated during the induction of fibroblast senescence by TGF-β1

**DOI:** 10.1038/s41598-022-17279-9

**Published:** 2022-07-26

**Authors:** May Leng Tan, E. Kenneth Parkinson, Lee Fah Yap, Ian C. Paterson

**Affiliations:** 1 Department of Oral and Craniofacial Sciences, Level 9, Postgraduate and Research Tower, Faculty of Dentistry, University of Malaya, 50603 Kuala Lumpur, Malaysia; 2 Centre for Immunobiology and Regenerative Medicine, Institute of Dentistry, Barts and the London School of Medicine and Dentistry, Queen Mary University of London, London, UK; 3 Oral Cancer Research & Coordinating Centre, Faculty of Dentistry, University of Malaya, Kuala Lumpur, Malaysia

Correction to: *Scientific Reports* https://doi.org/10.1038/s41598-020-79789-8, published online 12 January 2021

The original version of this Article contained errors in the Acknowledgments section.

“This research was funded by a University of Malaya Frontier Research Grant (FG001-17AFR) and a Fundamental Research Grant Scheme (FRGS) award (FP001-2018A) from the Ministry of Higher Education Malaysia.”

now reads:

“This research was funded by a Fundamental Research Grant Scheme award (FRGS/1/2018/SKK08/UM/01/2) from the Ministry of Higher Education Malaysia and a University of Malaya Frontier Research Grant (FG001-17AFR).”

The original Article has been corrected.

