# Supplementary material for: Autophagy is deregulated in cancer-associated fibroblasts from oral cancer and is stimulated during the induction of fibroblast senescence by TGF-β1
Source: Sci Rep. 2021 Jan 12;11:584. doi: 10.1038/s41598-020-79789-8 (PMC7804411; doi:10.1038/s41598-020-79789-8)

Autophagy is deregulated in cancer-associated fibroblasts from oral cancer and is stimulated during the induction of fibroblast senescence by TGF-β1

May Leng Tan[1], E Kenneth Parkinson[2], Lee Fah Yap[1] and Ian C Paterson[1,3,*]

[1]Department of Oral and Craniofacial Sciences, Faculty of Dentistry, University of Malaya, Kuala Lumpur, Malaysia
[2]Centre for Immunobiology and Regenerative Medicine, Institute of Dentistry, Barts and the London School of Medicine and Dentistry, Queen Mary University of London, UK
[3]Oral Cancer Research & Coordinating Centre, Faculty of Dentistry, University of Malaya, Kuala Lumpur, Malaysia

a

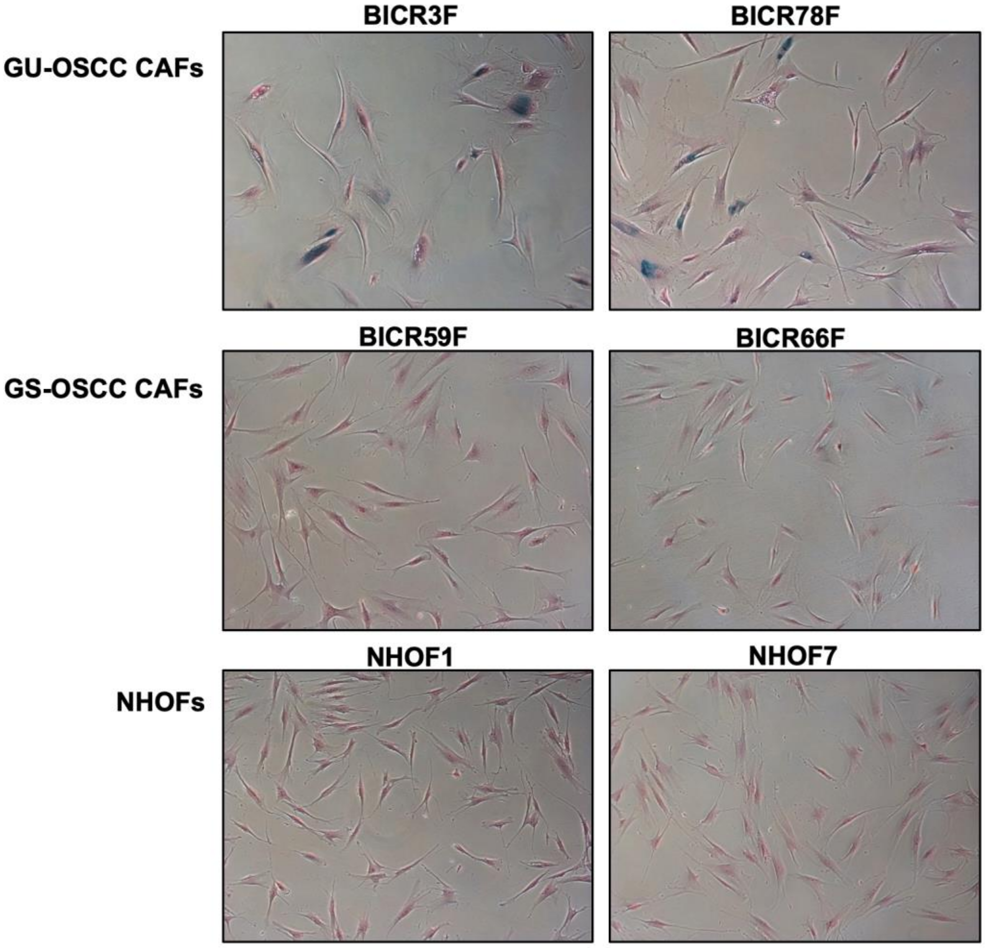

b

% of SA-β-gal

**GU-OSCC CAFs**	BICR3F	63.14
	BICR31F	66.41
	BICR63F	48.79
	BICR78F	49.66
**GS-OSCC CAFs**	BICR59F	9.38
	BICR66F	19.08
	BICR69F	16.53
	BICR73F	26.18
**NHOFs**	NHOF1	4.35
	NHOF2	2.22
	NHOF7	7.90

Supplementary Figure S1. Senescent phenotypes of normal oral fibroblasts and CAFs. (**a**) CAFs from GU-OSCCs displayed characteristics of senescent cells such as dark blue staining of SA-β-gal, enlarged cell size, hypertrophy and flatten cell morphology as observed by light microscopy. (**b**) The percentages of SA-β-gal positive cells in fibroblast cultures of GU-OSCCs, GS-OSCC and normal oral mucosa (NHOFs).

a

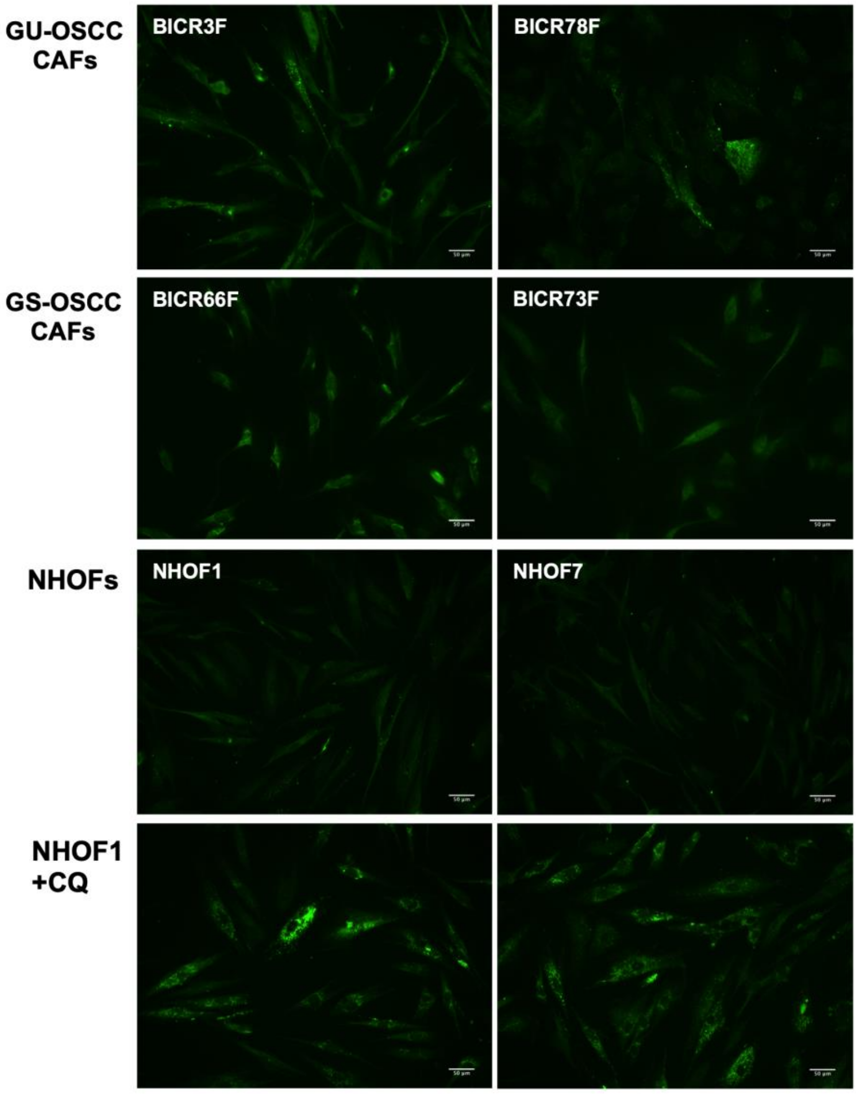

b

LC3B puncta per cell

**GU-OSCC CAFs**	BICR3F	40.25
	BICR31F	35.00
	BICR63F	6.89
	BICR78F	24.19
**GS-OSCC CAFs**	BICR59F	3.85
	BICR66F	2.48
	BICR69F	6.43
	BICR73F	1.98
**NHOFs**	NHOF1	2.74
	NHOF7	2.06

Supplementary Figure S2. Immunofluorescence staining of endogenous LC3B in oral fibroblasts. (**a**) Immunofluorescence staining showed more LC3B puncta in CAFs from GU-OSCCs. Cultured oral fibroblasts were fixed and stained with anti-LC3B antibody. LC3B punta were more evidently observed in CAFs from GU-OSCCs as compared to CAFs from GS-OSCCs and normal oral fibroblasts. Normal oral fibroblasts treated with CQ (5 μM, 24 hours) were used as a positive control. Scale bar indicates 50 μm. (**b**) Average number of LC3B puncta per cell in fibroblast cultures of GU-OSCCs, GS-OSCC and normal oral mucosa (NHOFs).

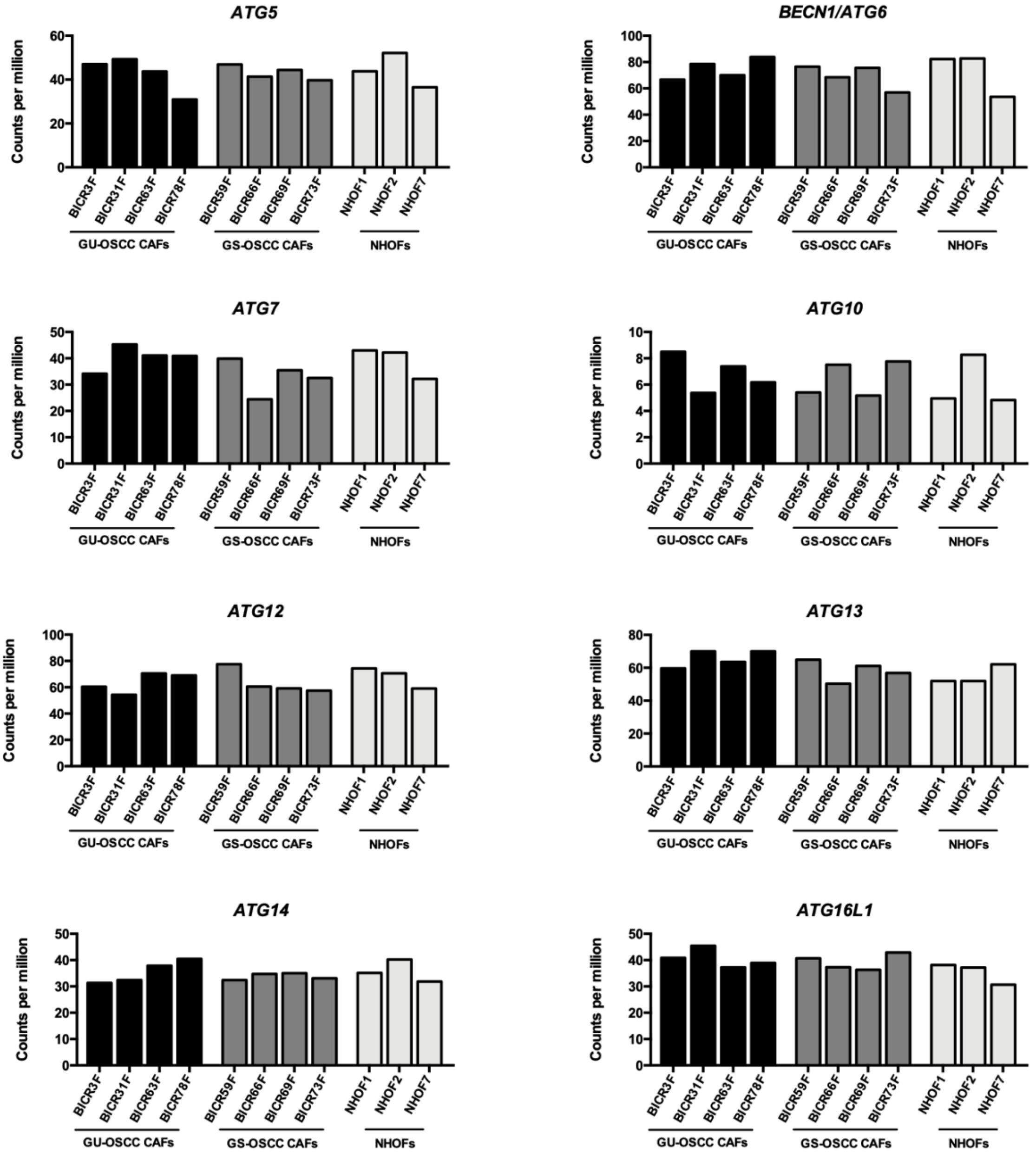

Supplementary Figure S3. Transcript quantification of autophagy-associated genes in CAFs and NHOFs with RNA sequencing. Bar charts displaying RNAseq data from a panel of CAFs from GU-OSCCs (n = 4), GS-OSCCs (n = 4) and normal fibroblasts (n = 3). The transcript abundance of each selected *ATG* gene - *ATG5*, *BECN1/ATG6*, *ATG7*, *ATG10*, *ATG12*, *ATG13*, *ATG14* and *ATG16L1* was expressed in counts per million.

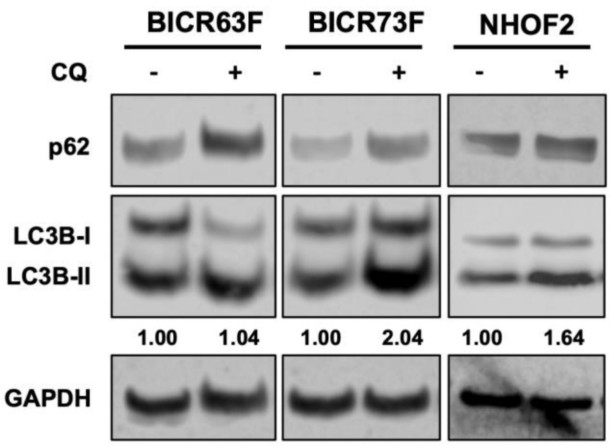

Supplementary Figure S4. Western blot of CAF from GU-OSCCs (BICR63F), CAF from GS-OSCCs (BICR73F) and NHOFs (NHOF2) with and without autophagic flux inhibitor, chloroquine (CQ) for LC3B protein turnover and p62. Densitometry values of LC3B-II are normalised to GAPDH and expressed relative to fibroblast treated with vehicle control (=1). Full-length blots are presented in Supplementary Fig. S8.

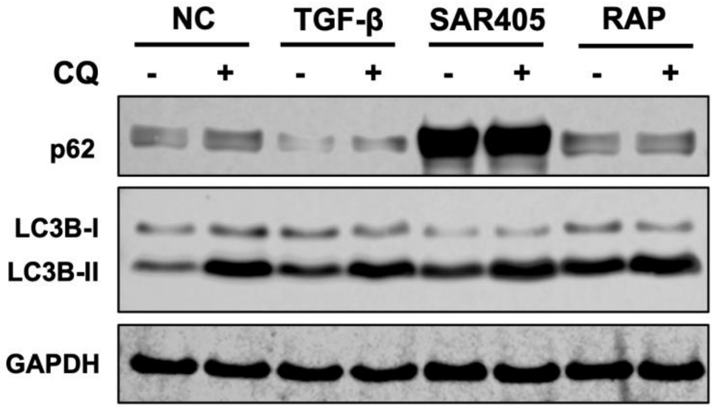

Supplementary Figure S5. To confirm the role of TGF-β and SAR405 on autophagy, autophagic flux assay were performed by treating cells with these compounds in the presence or absence of the autophagic flux inhibitor, chloroquine (CQ). Normal oral fibroblasts were treated with TGF-β (4 ng/mL) or SAR405 (10 μM) for 120 hours with or without CQ (20 μM, 6 hours). Negative control (NC) was left untreated and fibroblasts treated with the autophagy inducer, rapamycin (RAP, 250 nM, 24 hours) was used as positive control. The blot shown is representative of two independent experiments that produced similar results. Full-length blots are presented in Supplementary Fig. S8.

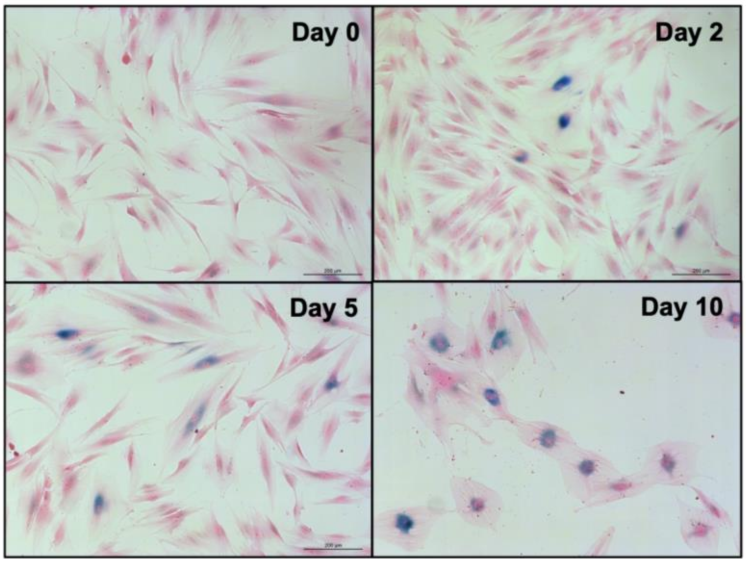

Supplementary Figure S6. Representative images of SA-β-gal staining in TGF-β1-treated normal oral fibroblasts over a period of 10 days. Senescent fibroblasts were characterised by dark blue staining of SA-β-gal. The cells underwent a morphological transformation typical of the senescent phenotype. Scale bad indicates 200 μm.

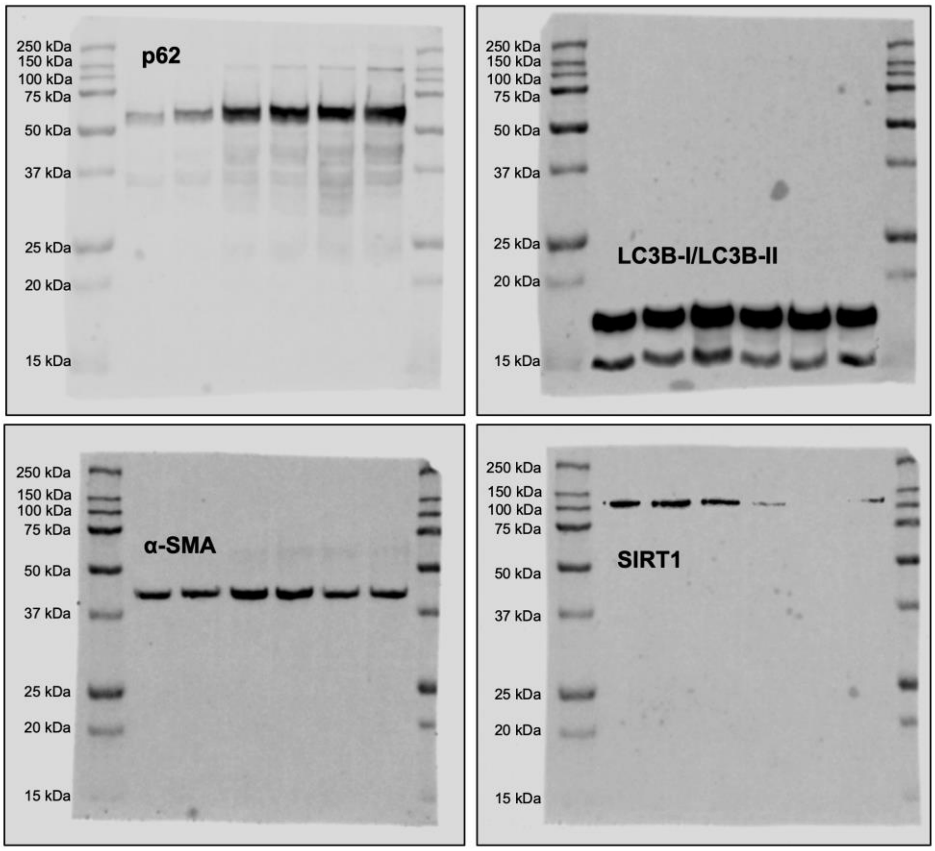

Supplementary Figure S7. Specificity of p62, LC3B, α-SMA and SIRT1 antibodies. Molecular weight markers are shown on the blots.

Supplementary Figure S8

Uncropped scans of Western blots. Molecular weight markers are shown on the blots.

Uncropped blots of Figure 1b

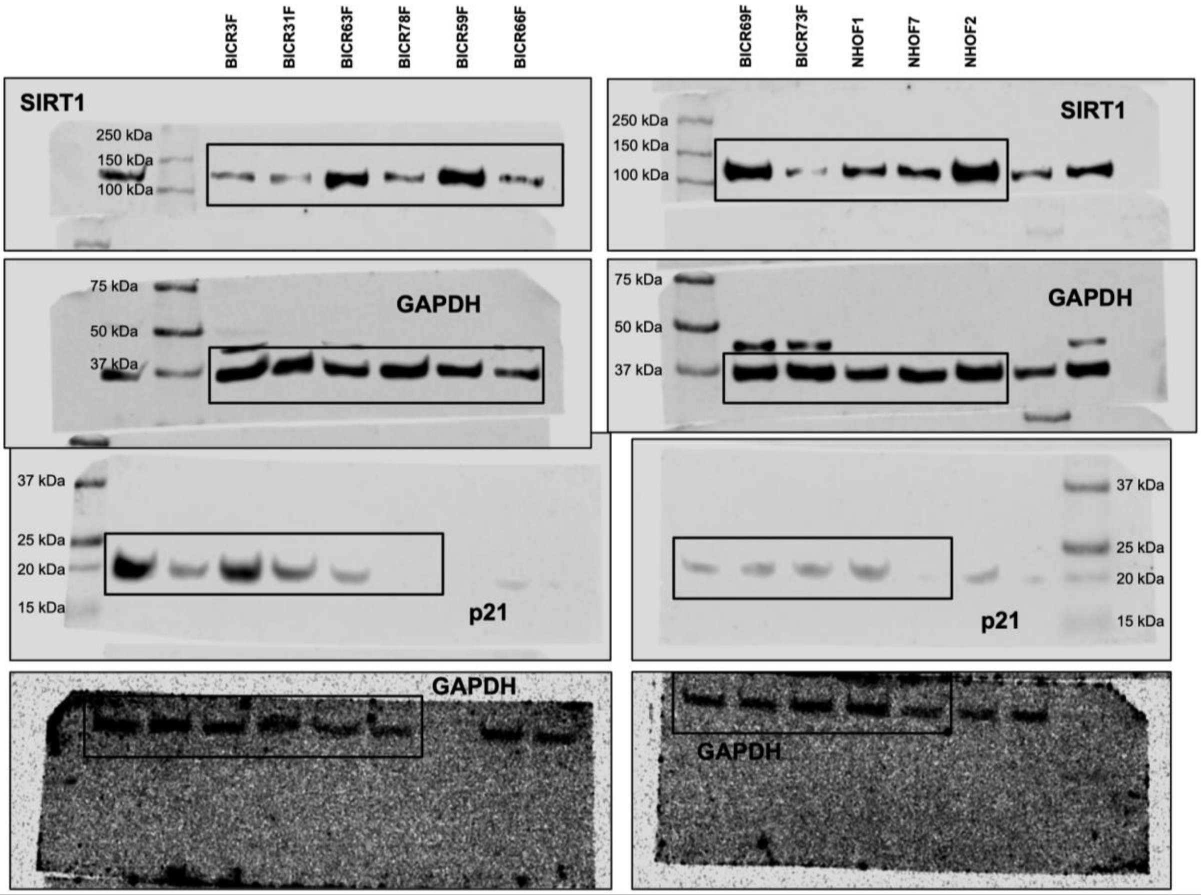

Uncropped blots of Figure 3b

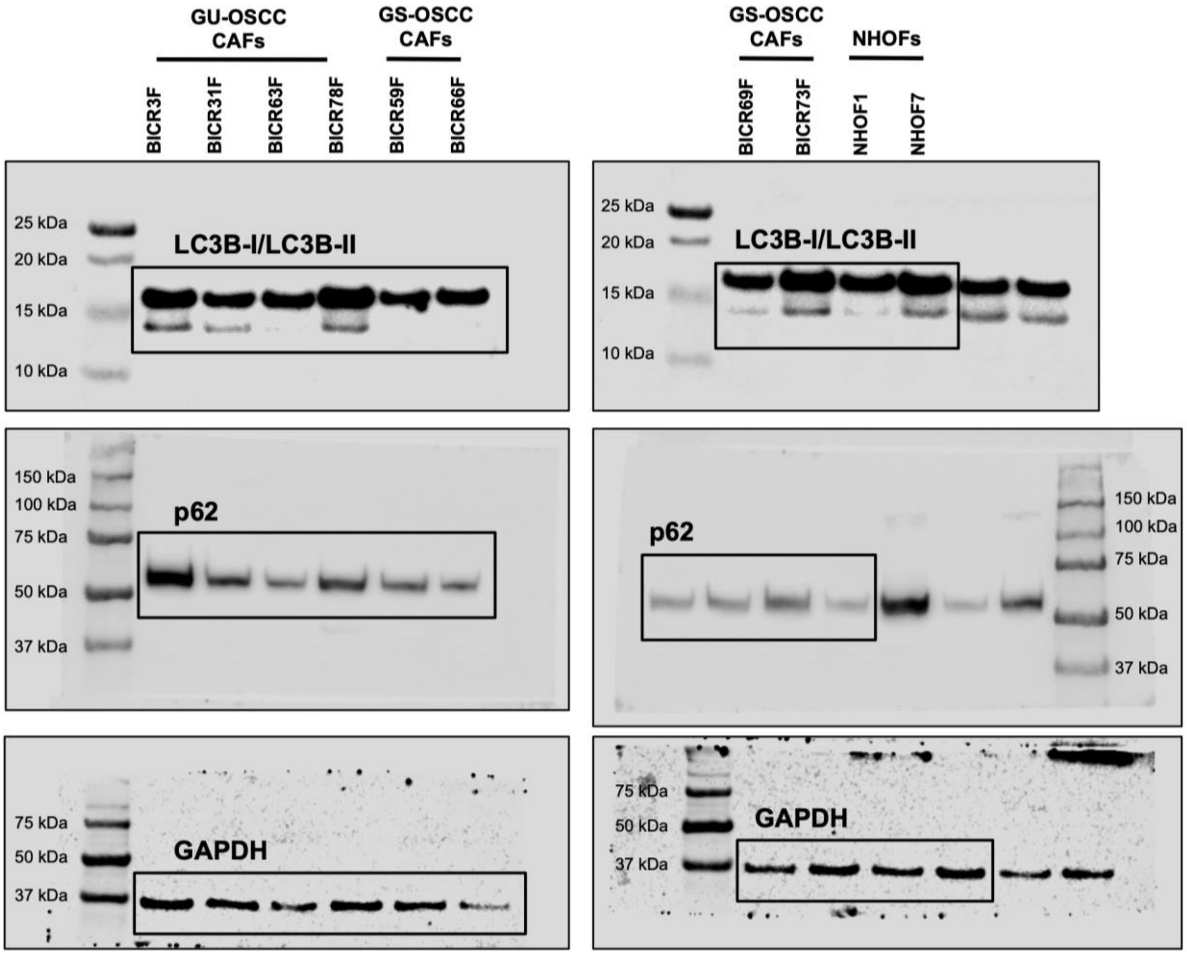

Uncropped blots of Figure 4a

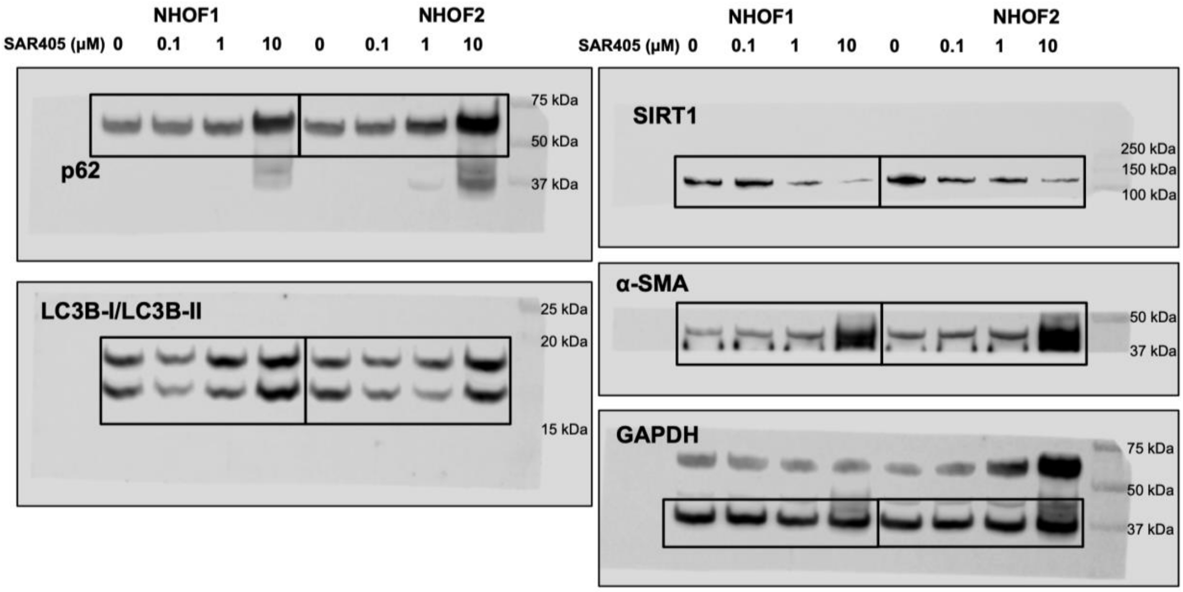

Uncropped blots of Figure 4b

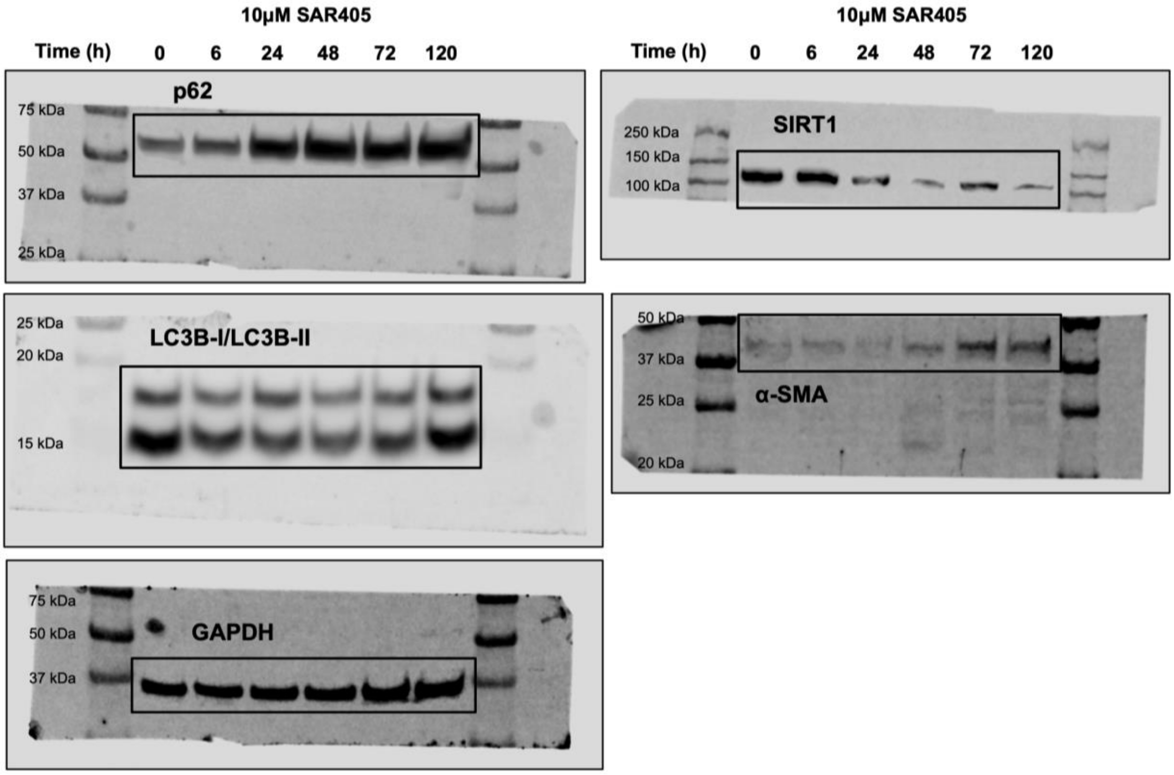

Uncropped blots of Figure 5a

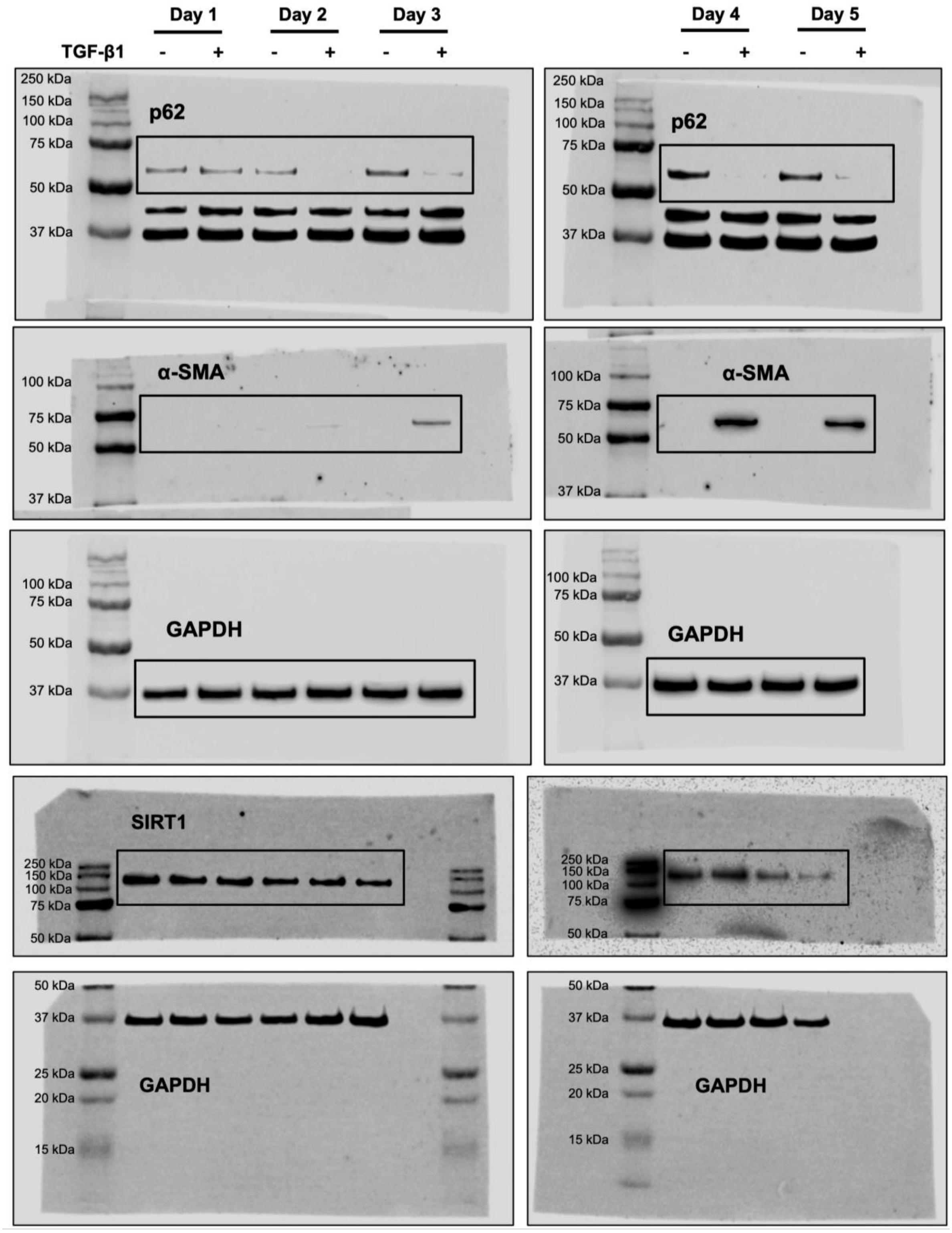

Uncropped blots of Figure 5c

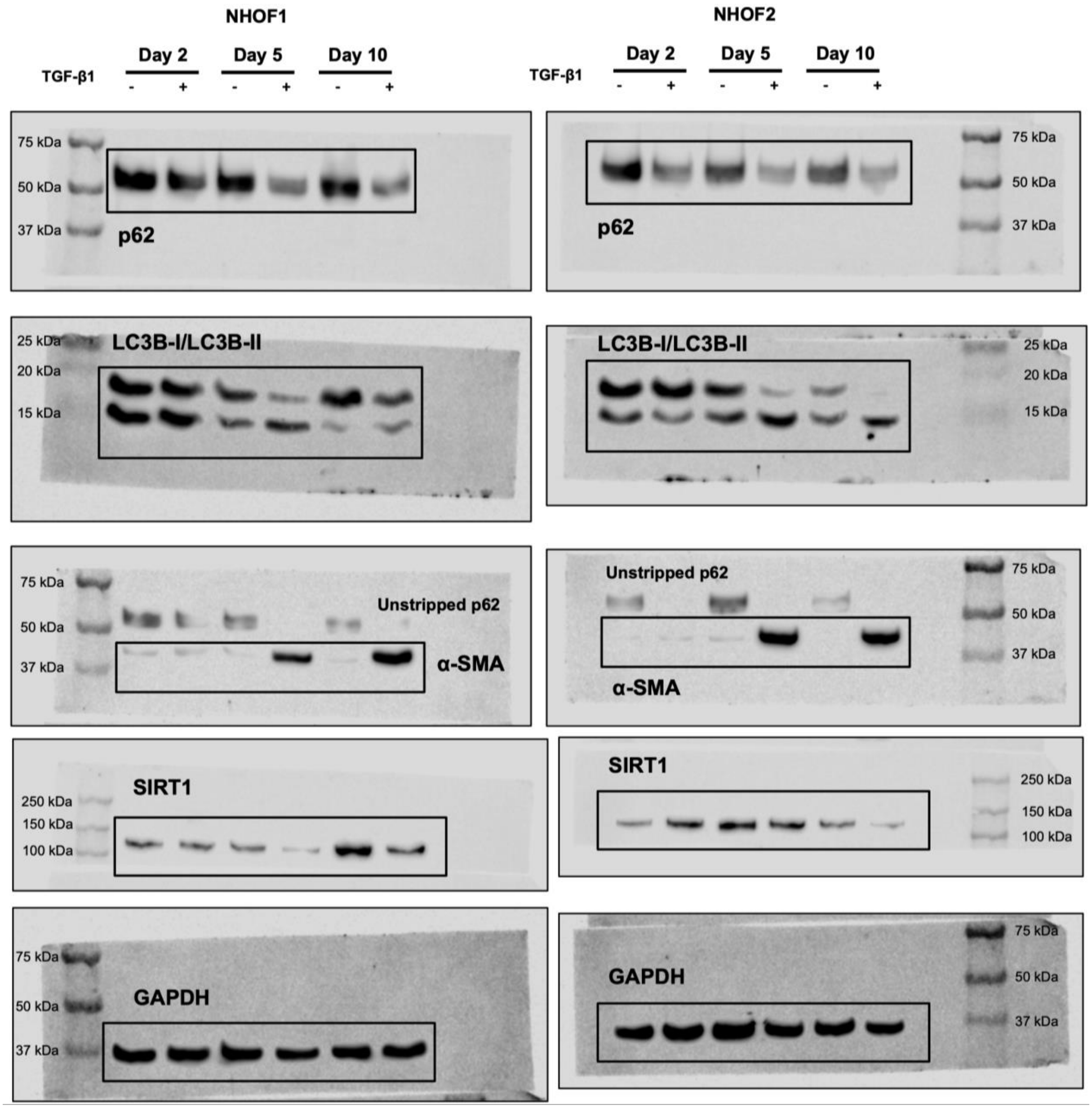

Uncropped blots of Figure 6a

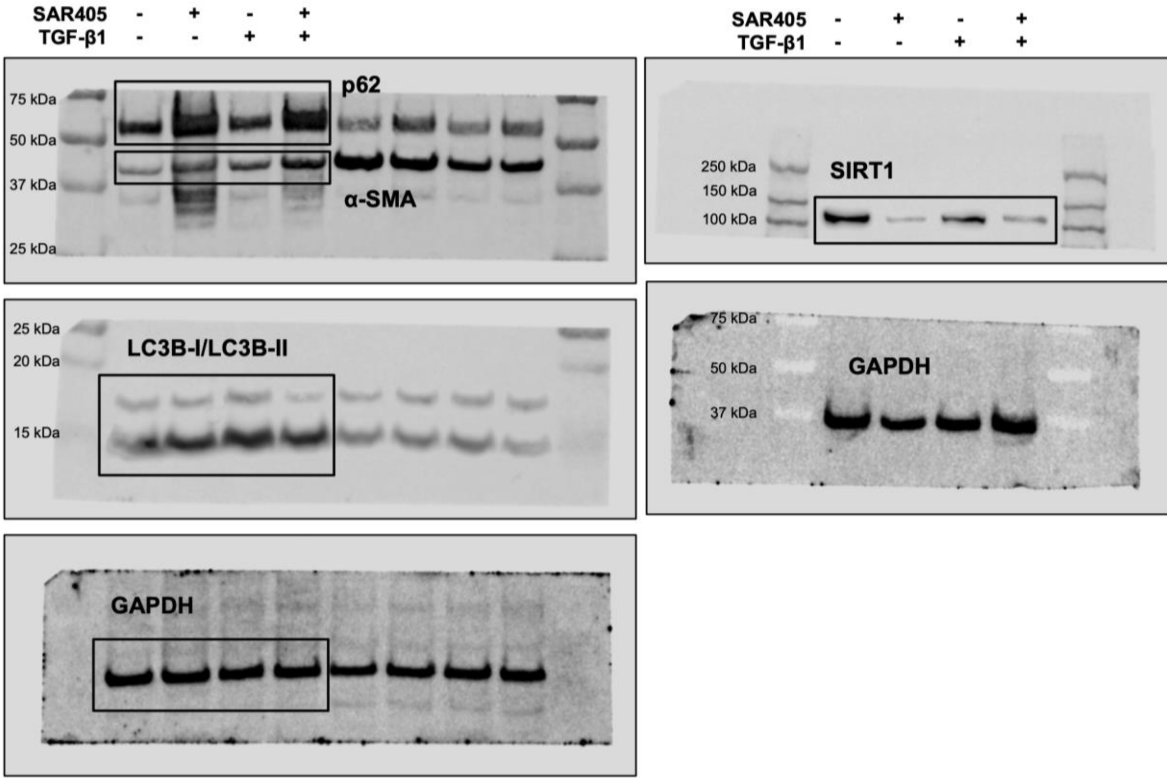

Uncropped blots of Supplementary Fig. S4

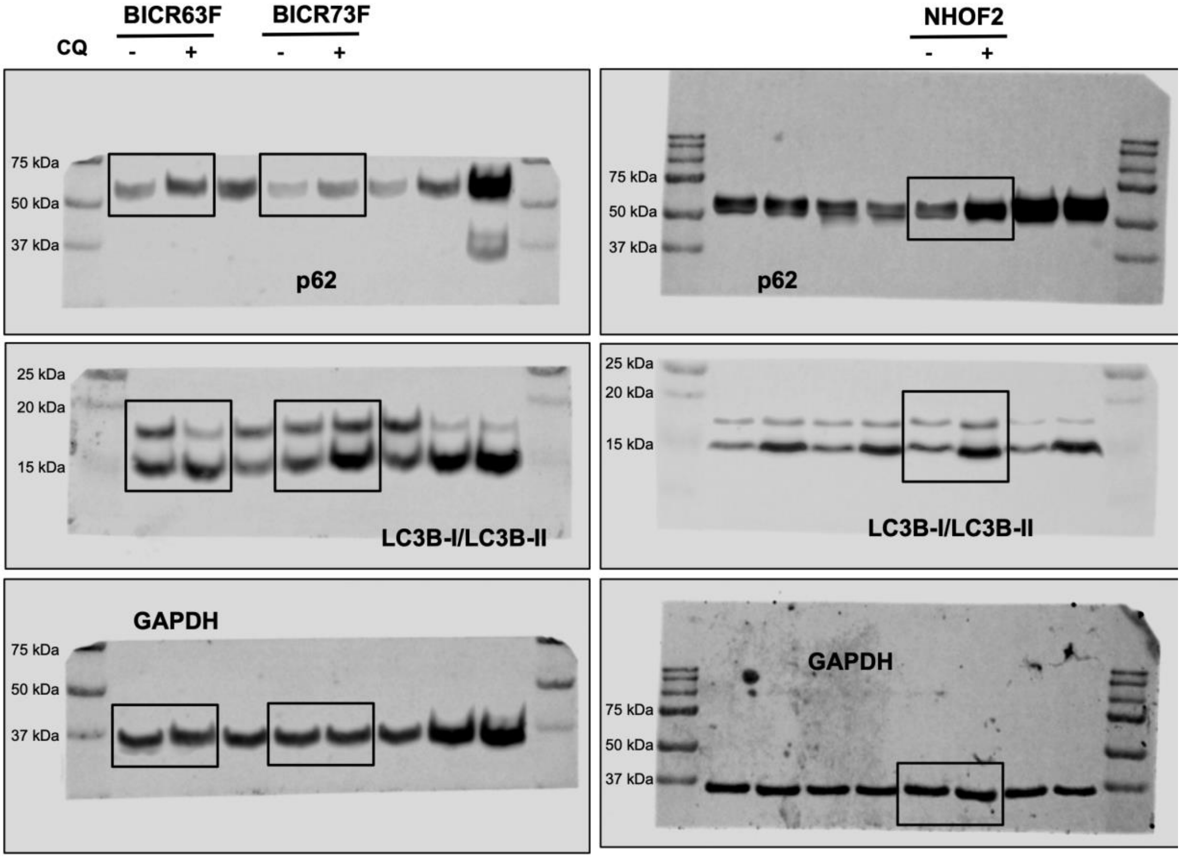

Uncropped blots of Supplementary Fig. S5

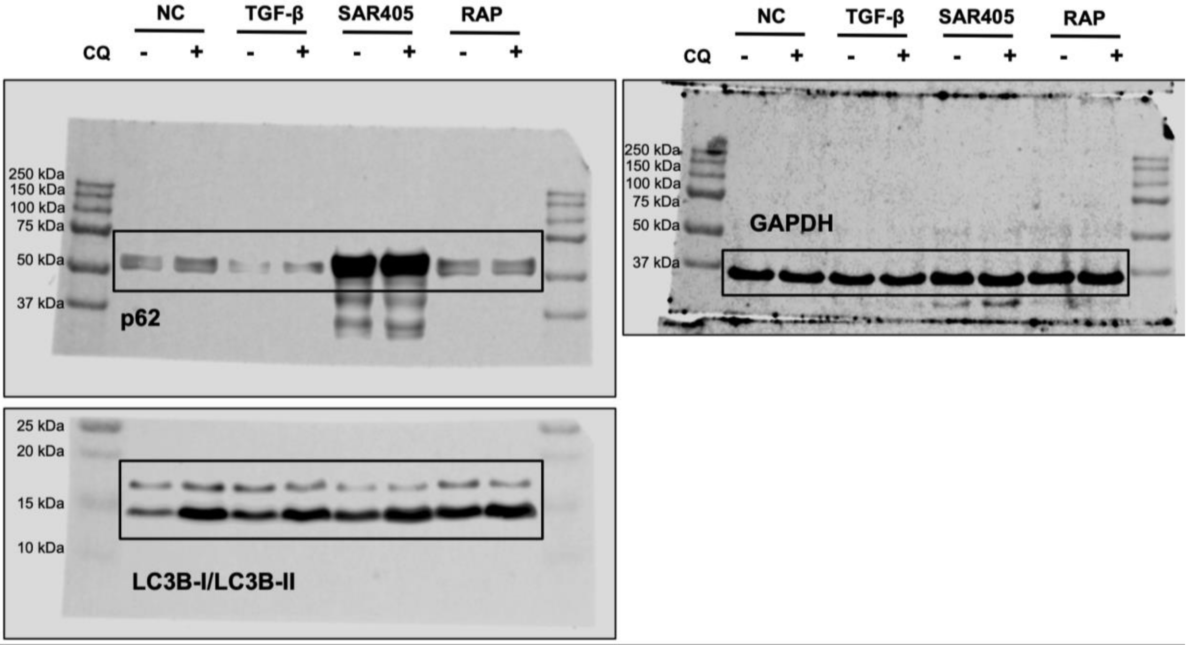

Supplement: Supplementary file 1 — Supplementary Figures. [file 41598_2020_79789_MOESM1_ESM.pdf]
